# Predictors of literacy in adulthood: Evidence from 33 countries

**DOI:** 10.1371/journal.pone.0243763

**Published:** 2021-03-11

**Authors:** Aki-Juhani Kyröläinen, Victor Kuperman

**Affiliations:** 1 Department of Linguistics and Languages, McMaster University, Hamilton, Ontario, Canada; 2 Department of Applied Linguistics, Brock University, St. Catharines, Ontario, Canada; University of Sao Paulo Medical School, BRAZIL

## Abstract

What makes a literate person? What leads to literacy gains and losses within and between individuals and countries? This paper provides new evidence that helps answer these questions. The present comparative analysis of literacy is based on large representative samples from the Survey of Adult Skills conducted in 33 countries, with 25–65 year old participants. We provide, for the first time, estimates of relative importance for a comprehensive set of experiential factors, motivations, incentives, parental influence, demands of workplace, and other predictors of influence. We sketch a configuration of factors that predicts an “ideal” reader, i.e., the optimal literacy performance. Moreover, we discover a pivotal role of the age effect in predicting variability between countries. Countries with the highest literacy scores are the ones where literacy decreases with age the most strongly. We discuss this finding against current accounts of aging effects, cohort effects and others. Finally, we provide methodological recommendations for experimental studies of aging in cognitive tasks like reading.

## Introduction

What makes a literate person? What leads to literacy gains and losses? These questions are at the forefront of current research in social sciences and humanities. To illustrate, a search for terms *reading comprehension* and *literacy* retrieved over 86,000 items out of the total of 90 million records in the comprehensive Web of Science reference database which covers all scientific fields. In other words, roughly one in a thousand papers produced by the entire scientific community in the last 40 years on any scholarly topic is related to issues of literacy.

Arguably, the ultimate goal of this line of research is to answer the question: what causes gains and losses in literacy. This goal transcends national, social, cultural, and linguistic boundaries and encompasses full ranges of age, gender, ability, and clinical conditions. A material help in achieving this goal comes from recent (since mid-1990s) international surveys of basic information-processing skills like literacy, numeracy, and problem solving in adolescents and adults [1, 2].

The present paper focuses on the most recent and comprehensive Survey of Adult Skills. This survey was launched in 2011 under the Programme for the International Assessment of Adult Competencies (PIAAC) guided by the Organization for Economic Cooperation and Economic Development [3] and has since collected rich cross-sectional datasets from adults (16–65 y.o.) in 39 countries of the world, most of them representing developed market economies. The data include direct assessments of information-processing skills—literacy, numeracy, and problem-solving—in the official language(s) of respective countries, as well as background demographic, social, and educational information, including information about skill use at the workplace and at home. Our present focus is on literacy which is defined in PIAAC as “understanding, evaluating, using, and engaging with written texts to participate in society, to achieve one’s goals, and to develop one’s knowledge and potential” [4]. Literacy tests directly rely on reading-related skills as they are likely to be used in real-life and workplace environments, that is, from the decoding of written words to the comprehension, interpretation, and evaluation of complex texts. An important characteristic of PIAAC testing is that its scores are valid and comparable between all cross-sectional national samples. With target sample sizes of N = 5,000 or more and the multi-stage sampling design, the samples are designed to be representative of the entire adult (16-65 y.o.) non-institutionalized population of each respective country. If a national survey was designed to oversample certain population groups (i.e., younger or older groups, or linguistic minorities), the overall sample size was increased accordingly [3]. This design and scope make PIAAC a valuable data source for pursuing the questions this paper begins with [5, 6].

Unsurprisingly, availability of PIAAC and similar surveys as data sources has generated a substantial boost in the literature on labor-market economics, sociology, education, and management (as indicated by the Web of Science citation analysis of bibliographic items containing “PIAAC” in their title). This literature establishes reading literacy as a foundational skill in a modern technological society. It describes profound economic, cultural, and social impacts of an inadequate literacy level on a person’s employability, income, well-being, health, and social engagement in the modern technological society and marketplace [7–9].

Perhaps the most important and disturbing finding in this literature is that a large share of the adult population falls short of the literacy competency needed for skilled workers in the modern society (under level 3 in the PIAAC classification of literacy levels, see below). This is true even for developed economies and among educated populations: e.g., over 50% on average in all countries that have participated in the PIAAC Survey are functionally illiterate, over 40% in Canada, and one in four university-educated and one in two college-educated students in the province of Ontario, Canada [10–13].

Against this background, it is surprising that cross-linguistic international literacy data from PIAAC have gone largely unnoticed in psychological and linguistic research centering on language use, cognitive development, learning, and skill acquisition [14]. To fill this lacuna, the present paper aims to analyze literacy data along with a large number of background variables from the PIAAC survey in search for robust predictors of literacy in 33 countries. Our goal is to identify the relative importance of an inclusive list of variables that predict different levels of literacy, within and across countries. In Analysis 1, we pursue a bird’s-eye characterization of predictors of literacy which glosses over variability between individual samples [15, 16]. Analysis 2 zooms in on specific factors that drive variability between countries. To anticipate the results, the effect of age on literacy turned out to be both important and puzzling. In Analysis 2, we introduce theoretical accounts linking age and dynamics of literacy skill and pit new evidence against these accounts.

This approach deviates from the common practice of individual survey-based studies to track the impact of a small number of predictors in a restricted subset of country samples (e.g., immigration status, age, cultural capital of the family, type of occupation or education, but see [17]). Our approach is also different from most existing cross-linguistic studies of reading skills [18, 19] that concentrate on beginning readers and often lack much of relevant background information on language users. While childhood is a critical period for developing the reading skill, we adopt the view of literacy acquisition and use as a lifelong learning process, i.e., “from womb to tomb” [15]. We believe that in an individual this process reflects their genetic and biological predispositions; the dynamics of opportunities, demands, and incentives afforded by the ever-changing environment; motivational factors; as well as the quantity and quality of practice with the skill [20–22]. The PIAAC data from participants representing several decades of adulthood enable us to focus on mechanisms and outcomes of this lifelong learning process. Our main interest is in the cognitive and linguistic factors that co-determine the distribution of literacy skill within and between countries.

In sum, we aim to offer new evidence on what makes a person literate across countries and languages, as well as provide some guidance to psychological research by pointing to the factors that warrant additional scrutiny and tighter experimental control in future research. Finally, we will highlight the practical implications of our findings, which may be of interest to social scientists and policy-makers.

## The present study

In line with several prior analyses [23, 24], we only consider here native-born speakers of the official language(s) of the country and the test administration. Immigrants are likely to have a divergent set of linguistic, educational, and workplace experiences and require a separate analysis [25, 26]. Also, we specifically concentrate on adults between 25 and 65 y.o. Adults over 65 were not part of the data collection in PIAAC (except for the USA sample) [5, 6]. We also excluded from analyses individuals below 25 y.o. who are likely to be undergoing a drastic change in their literacy skills as many of them are taking part in formal education: for justification see e.g., [23, 24].

### Literacy and its predictors

The dependent variable of this study is literacy score. PIAAC assesses literacy in the tasks that test comprehension, evaluation and integration of words, sentences and texts in authentic information-processing contexts [4, 27]. Some texts are continuous (tapping into prose literacy) and some discontinuous (document literacy); several genres of texts are used also (narration, exposition, persuasion). A typical assignment might require understanding a written text mimicking a web-page and navigating to the required piece of information using menus and hyperlinks (for a demo see https://www.oecd.org/skills/piaac/onlineassessmment/demo/). The majority of participants across countries took the digital version of the literacy test. A printed version was also available to individuals uncomfortable with using computer technology: these individuals also completed a separate test of reading skill [28], which is not discussed here.

The literacy scale ranges from 0 to 500 points, with the observed range across samples from 0 to 464. PIAAC has identified five discrete achievement levels for literacy, with level 3 (276–325 points) or higher deemed sufficient to operate as a skilled worker and participate in social and cultural life in the modern information-based economies [4].

Earlier reports have identified a large number of background variables that influence the distribution of literacy scores. We begin their exposition with variables that tap into extrinsic motivation to improve one’s literacy skill (i.e., driven by external incentives like succeeding in school or at work), and proceed to those related to intrinsic motivation (e.g., self-driven by the interest in reading in its own right), and finally control variables [29, 30]. The detailed description of the derived variables (READWORK, NUMWORK, LEARNATWORK, NUMHOM and READHOME) used in this study is provided in S3 Section in S1 File.

#### Formal education

Formal educational attainment after the mandatory schooling is known to strongly correlate with literacy levels [31–33]. Robustly, a greater number of years of education or a higher vocational, professional or academic degree comes with higher and long-lasting achievements in the literacy domain [34]. Reder argues that the correlation reflects two overlapping processes: literacy development and literacy selection [35, 36]. Formal education often requires intensive engagement and practice in information-processing skills, which also provides extrinsic motivation and incentives for development of those skills. Longitudinal studies of post-secondary students confirm a gradual development in their literacy and its component skills throughout the years of education [37]. Yet formal education also engenders selective filtering: institutional acceptance and retention decisions filter out less proficient individuals; moreover, individuals with perceived lower skills may self-select not to pursue non-mandatory advanced education. Thus, as with most other predictors, the relationship between formal education and literacy is reciprocal: we discuss this issue below. In this study, we use the PIAAC’s 6-level ISCED classification of formal education levels.

#### Reading and numeracy at work

A central contribution of the PIAAC survey is its scrutiny of skills in use at one’s workplace. Similar to formal education, holding a job with substantial demands for information-processing skills, including literacy, is expected to enhance an individual’s literacy level. At the same time, self-selection of prospective workers and selectivity of the marketplace may both be based on the individual’s readiness to perform at the required literacy skill level. We examine contributions of workplace literacy and learning-on-the-job by considering three PIAAC variables: an index of use of reading skills at work (READWORK) and numeracy skills at work (NUMWORK), and of the perceived amount of learning that one undergoes at work (LEARNATWORK) [38]. Numeracy is relevant because it is known to share variance with literacy both in the early development of these two skills and in adults, and yet it has substantial unique variance as well [39, 40]. This contrasts with the writing skill, which we did not consider because of its high collinearity with reading.

#### Informal education

Lifelong learning can be pursued in a variety of ways, some involving informal education (e.g., delivered via after-school programs, online courses, community-based organizations, museums, libraries, or at home). Pursuit of informal education relies on intrinsic motivation for learning, and has also been shown to increase literacy levels, through a sustained practice of reading and understanding texts [41–43]. We use PIAAC variable NFE12, which indicates whether an individual engaged in any kind of informal education in the last 12 months. This question was a binary choice.

#### Reading and numeracy at home

Reading for pleasure has long been identified as an important and lasting predictor of reading development and proficiency in children, adolescents and adults (see e.g., reviews by [38, 44, 45]). Engaging in this self-driven, intrinsically motivated activity leads to a robust and accumulating advantage in literacy skills [46] and, in a reciprocal way, more literate individuals will more readily engage in reading which strengthens their exposure to print. This “Matthew effect”, which labels an upward spiral for better readers and a downward spiral for poorer ones [22], is a hallmark of reading and literacy skill development. To quantify this effect, we consider derived PIAAC estimates of the amount and habits of reading done at home (variable READHOME) as well as the use of numeracy at home (NUMHOME).

Broadly speaking, all independent variables listed above are at least partly related to literacy because they modulate the amount, quality, or intensity of reading practice that a person engages in. Psychological literature terms this practice as “exposure to print” and has demonstrated its positive correlation with proficiency in reading comprehension among children, adolescents and young adults of the college age [47–49].

#### Age

Importantly, exposure to print also continues to accumulate with advancing age, and as such should lead to a gradual increase in literacy. Age also comes with a continuous lifelong accumulation of knowledge as seen in vocabulary growth [50–53]. As we will discuss at length in Analysis 2, age stands out from the range of other independent variables in several ways. Here we confine ourselves to a frequently made observation that age has a negative effect on literacy and other information-processing skills, with younger adults showing higher scores than older ones [54–56]. In this study, we made use of the age variable that was discretized into eight five-year bins (AGE5GLFS), ranging from 25 to 65 y.o.

#### Control variables

Additionally, we considered variables of demonstrated or potential importance for literacy performance. One group of controls is parental resources, i.e., an indirect index of how supportive home environment is for developing literacy skills and pursuing education. We included measures that prior literature deemed influential [23, 24, 57–61]: education level of the mother and father, whether or not parents were immigrants, and the number of books in the household where the responder grew up [5, 6]. We also included an occupational status of a person (EDWORK) with values indicating whether a person holds a job; is in education; both holds a job and is enrolled in formal education; is not employed but getting trained for job; or is not employed or in training. Health status (HEALTH), ranging from 1 “excellent” too 5 “poor” and gender (GENDER_R) were considered as well.

## Methods

As a source of the PIAAC data, we used the publicly available files from 35 countries (https://www.oecd.org/skills/piaac/data/). However, two national samples were removed from the analysis. First, the Russian sample did not report several variables critical for our analysis, e.g., whether an individual was born in Russia and whether an individual’s home language is the official language of this country. Second, after trimming described below, the Singapore sample was represented by too few datapoints (458), which could lead to overfitting of regression models to the data given a large number of predictors.

The following data-processing and trimming steps were applied. As motivated in the Introduction, we only considered individuals who were born in the country of test administration and were native speakers of one of the languages in which the test was taken. We further removed individuals between 16 and 24 y.o. (see The Present Study section for rationale) as well as individuals with missing values for education and occupational status.

Sizes of the final 33 national subsamples with 25–65 y.o. adults are shown in Table 1: they ranged from 1,796 (Kazakhstan) to 15,415 (Canada) respondents and represented an average 67% (*SD* = 11) of the original sample sizes. The vast majority of removed cases were due to our age and residence/language restrictions. Table 1 further reports the mean, SD, and rank of the literacy score of each national subsample, as well as original national sample size.

**Table 1:** Size of the full PIAAC samples and native-born native-language subsamples, and mean, SD, and rank of the literacy scores in these subsamples.

Country	N	N Subsample	*M* Score	*SD* Score	Rank
bel	5463	3499	277.29	44.76	11
can	26683	14910	279.05	47.31	8
chl	5212	3950	215.77	52.62	31
cyp	5053	3250	270.37	40.35	18
cze	6102	4302	273.22	40.34	15
deu	5465	3490	273.64	45.61	14
dnk	7328	4838	274.81	43.87	13
ecu	5702	3973	193.42	50.47	33
esp	6055	3914	253.69	47.83	26
est	7632	4575	279.20	43.79	7
fin	5464	4194	288.95	47.80	2
fra	6993	4692	265.75	45.97	20
gbr	8892	6423	277.97	46.74	10
grc	4925	3843	253.67	46.35	27
hun	6149	4926	263.48	45.36	23
irl	5983	4069	266.90	46.88	19
isr	5538	1934	261.59	57.07	24
ita	4621	3530	251.00	43.39	28
jpn	5278	4314	296.47	39.88	1
kaz	6050	1622	250.60	39.22	29
kor	6667	5315	270.69	40.20	17
ltu	5093	3796	265.07	40.83	21
mex	6306	4587	220.62	47.17	30
nld	5170	3684	288.39	44.63	4
nor	5128	3340	284.72	42.09	6
nzl	6177	3230	285.18	45.62	5
per	7289	5159	195.14	50.52	32
pol	9366	4728	264.29	48.25	22
svk	5723	3893	277.02	37.33	12
svn	5331	3757	257.30	47.43	25
swe	4469	2855	288.55	42.27	3
usa	7921	3359	278.20	47.04	9

One of the attractive features of PIAAC and similar surveys is that their samples are weighted and can be used to make inferences about populations of entire countries. This is achieved with the help of weights that enable each observed respondent to stand for a larger segment of the population. To estimate contributions of predictors to literacy scores, we used ordinary least squares regressions with Jackknife Repeated Replication weights that correct for the complex designs of PIAAC samples which vary from country to country [3]. The appropriate regression functions are implemented in the package instvy that is designed specifically for the PIAAC data [62] and is provided in the statistical platform R 3.6.1 [63]. Specifically, the function implements a procedure where a regression model for each national sample uses weights to predict literacy scores for the entire population based on a set of predictors (see above). All the predictors were sum-coded prior to the analysis. For each country, the fitted values were averaged while their variance was corrected due to imputation [3].

We acknowledge that relationships between literacy and predictors that we consider present a complex network of direct, indirect and reciprocal effects, which may warrant use of sophisticated analytical techniques (e.g., path analysis, Structural Equation Models, or random forests, [61, 64–66]. For the present purposes, we glossed over this complexity and confined ourselves to weighted linear multiple regression models.

To reiterate, our goal is to establish the relative importance of virtually all predictors of literacy proposed in the literature against a very large international data pool. Analysis 1 concentrates on commonalities in literacy development and performance across countries and languages. Analysis 2 complements this approach by determining which predictors differentiate between countries and how they do so.

## Analysis 1: What predicts literacy?

### Results

**Fig 1:**
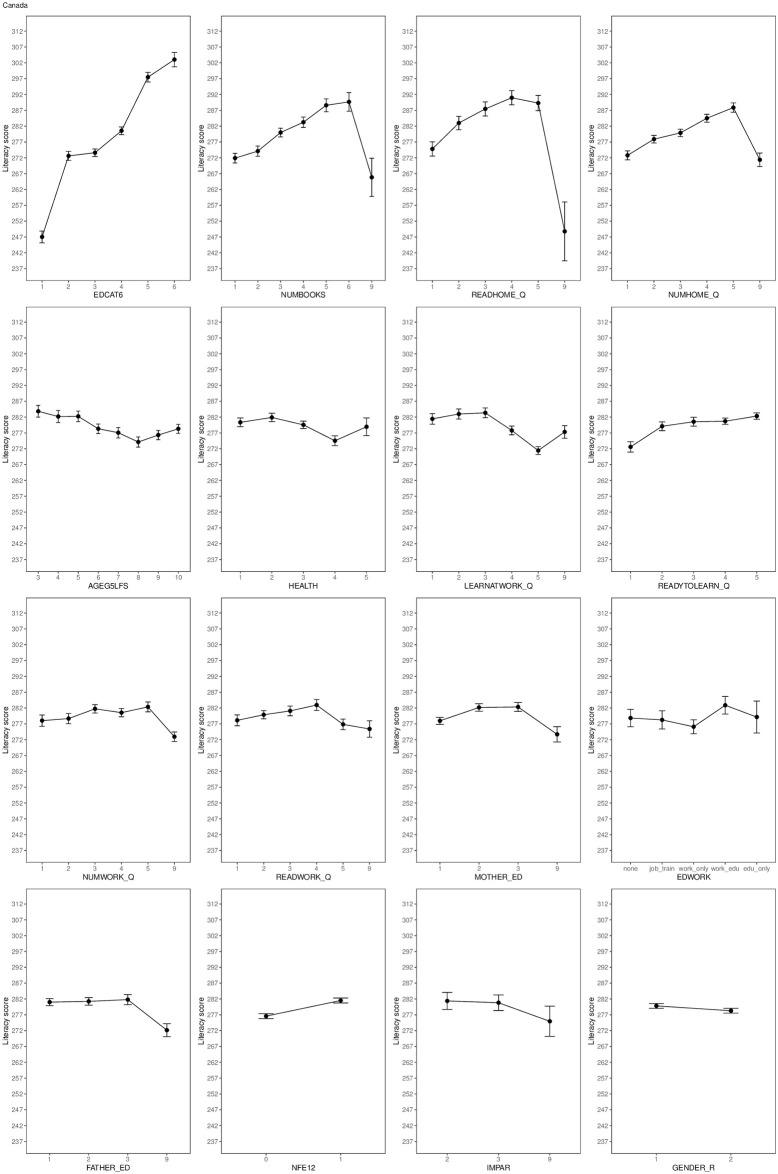
Partial effects of predictors on literacy scores in the Canadian sample. Error bars represent the 95% confidence interval. Value “9” stands for missing or undefined responses excluding the predictor age (AGEG5LFS).

This analysis determines relative roles of predictors of literacy across national samples. Country-specific regression models fitted to literacy scores explained between 17% (Cyprus) and 51% (Israel) of variance, with a median of 40% (IQR 12%), see Table 2. The functional form and direction of each predictor’s effect were fairly consistent across countries. For illustration, we visualize partial effects of all predictors to literacy scores in the largest national sample, Canada (see Fig 1). Plots with partial effects for all countries’ samples are provided in S2 Section present in S1 File.

**Table 2:** Aggregated effect sizes of literacy predictors. Estimated as partial eta-squared (in percent) and standardized effect (in units of *SD*). Also reported are partial *η*^2^ estimates of predictors by country; aggregated and country-specific R^2^ of regression models.

Predictor	eta2_median	eta2_IQR	aut	bel	can	chl	cyp	cze	deu	dnk	ecu	esp	est	fin
EDCAT6	6.89	3.32	5.13	10.51	11.03	9.48	7.64	5.81	8.56	6.31	2.1	7.38	5.38	7.03
NUMBOOKS	1.74	1.04	2.3	1.07	1.78	0.99	0.19	2.09	3.09	1.93	1.45	2.51	2.15	1.74
READHOME_Q	1.31	0.87	0.38	0.54	1.48	1.36	0.66	0.88	1.47	2.98	0.76	1.31	1.04	1.63
NUMHOME_Q	1.2	0.9	0.54	1.11	1.33	0.28	0.23	2.06	1.2	1.48	0.18	0.86	1.77	1.29
AGEG5LFS	1.06	1.63	2.23	2.09	0.69	1.21	0.31	1.69	2.94	4.44	0.37	2.36	0.39	5.44
HEALTH	0.73	0.82	1.39	0.27	0.29	1.53	1.4	1.25	0.53	1.16	0.55	1.57	0.45	0.27
LEARNATWORK_Q	0.65	0.68	0.63	0.65	1.18	0.79	0.21	0.87	1.32	0.64	0.1	0.38	0.7	0.93
NUMWORK_Q	0.45	0.34	0.7	0.75	0.43	0.55	0.11	0.19	1.17	0.66	0.38	0.32	0.2	0.38
READYTOLEARN_Q	0.4	0.43	0.26	0.08	0.49	0.61	0.75	0.25	0.7	0.11	1.37	0.4	0.13	0.23
READWORK_Q	0.34	0.21	0.34	0.31	0.38	0.31	0.64	0.84	0.13	0.58	0.86	0.16	0.25	0.28
EDWORK	0.3	0.39	0.55	0.16	0.2	0.37	0.26	0.37	0.6	0.49	0.13	0.83	0.81	0.38
MOTHER_ED	0.27	0.2	0.4	0.71	0.32	0.23	0.03	0.11	0.53	0.23	0.21	0.06	0.42	0.24
FATHER_ED	0.21	0.14	0.05	0.05	0.26	0.14	0.09	0.63	0.33	0.22	0.17	0.05	0.2	0.22
NFE12	0.08	0.18	0.32	0.01	0.32	0.03	0.02	0.01	0.16	0.08	0.35	0.06	0.11	0.07
GENDER_R	0.06	0.08	0.08	0.24	0.05	0.59	0.06	0.01	0.01	0.01	0.08	0.3	0.01	0.02
IMPAR	0.06	0.09	0.11	0.05	0.03	0.09	0.1	0.19	0.02	0.12	0.15	0.07	0.59	0.04
R2	0.4	0.12	0.37	0.42	0.42	0.45	0.17	0.3	0.46	0.43	0.23	0.44	0.31	0.45

We estimated effect sizes and relative importance of predictors in each model with the help of partial *η*^2^. This index measures a predictor’s independent contribution to explaining variance in literacy scores when variance associated with other predictors is partialled out. Partial *η*^2^ values are comparable across models and are reported jointly.

Tables 2–4 report partial *η*^2^ effect-size estimates for each country, as well as the median and IQR of each effect size across all countries. Variables are presented in Table 2 and are discussed below in decreasing order of their median *η*^2^ aggregated across all countries. We further tested a large number of two-way interactions between education, age and other predictors: many of them have reached the nominal significance level (0.05) thanks to a very high number of observations, but all had negligibly small effect sizes (partial *η*^2^ < 0.1%). We do not discuss these interactions further.

**Table 3:** Cont.: Aggregated effect sizes of literacy predictors. Estimated as partial eta-squared (in percent) and standardized effect (in units of *SD*). Also reported are partial *η*^2^ estimates of predictors by country; aggregated and country-specific R^2^ of regression models.

Predictor	eta2_median	eta2_IQR	fra	gbr	grc	hun	irl	isr	ita	jpn	kaz	kor	ltu	mex
EDCAT6	6.89	3.32	6.79	6.89	2.45	6.89	9.27	5.72	3.63	7.5	1.9	6.94	1.93	4
NUMBOOKS	1.74	1.04	2.25	2.45	1.42	2.02	1.64	0.49	1.52	1.49	2.06	0.66	0.55	0.69
READHOME_Q	1.31	0.87	1.22	1.27	0.55	1.44	4.26	4.09	1.33	1	2.19	1.38	1.18	3.38
NUMHOME_Q	1.2	0.9	1.2	1.66	0.63	1.22	0.56	0.6	1.4	0.72	1.72	0.48	1.08	1.06
AGEG5LFS	1.06	1.63	2.17	0.42	0.93	0.76	0.46	0.89	0.95	7.36	0.92	3.64	1.06	1.24
HEALTH	0.73	0.82	0.75	0.96	0.21	1.4	0.58	1.1	0.1	0.43	0.23	0.21	0.48	1.44
LEARNATWORK_Q	0.65	0.68	0.55	1.36	0.35	1.08	0.4	1.09	0.82	0.56	1.93	0.39	1.05	0.31
NUMWORK_Q	0.45	0.34	0.58	0.76	0.27	0.58	0.43	0.65	0.48	0.46	0.58	0.24	0.38	0.13
READYTOLEARN_Q	0.4	0.43	0.41	0.2	0.33	0.05	0.19	1.78	0.52	0.37	0.24	0.68	0.62	0.57
READWORK_Q	0.34	0.21	0.46	0.16	0.11	0.25	0.2	0.89	0.4	0.33	1.47	0.13	0.25	0.68
EDWORK	0.3	0.39	0.27	0.25	0.63	0.17	0.19	1.75	0.12	0.66	0.78	0.5	0.1	0.54
MOTHER_ED	0.27	0.2	0.39	0.62	0.5	0.29	0.26	1.53	0.1	0.39	0.22	0.14	0.23	0.29
FATHER_ED	0.21	0.14	0.16	0.29	0.16	0.29	0.21	0.55	0.2	0.02	0.49	0.14	0.24	0.24
NFE12	0.08	0.18	0.05	0.71	0.1	0.53	0.08	0.07	0.33	0.02	0.02	0.22	0.13	0.06
GENDER_R	0.06	0.08	0.01	0.07	0.13	0.1	0.69	0.18	0.05	0.01	0.02	0.34	0.02	0.06
IMPAR	0.06	0.09	0.04	0.12	0.02	0.1	0.07	0.31	0.02	0.05	0.13	0.05	0.06	0.05
R2	0.4	0.12	0.4	0.4	0.18	0.49	0.4	0.51	0.32	0.36	0.2	0.38	0.25	0.35

**Table 4:** Cont.: Aggregated effect sizes of literacy predictors. Estimated as partial eta-squared (in percent) and standardized effect (in units of *SD*). Also reported are partial *η*^2^ estimates of predictors by country; aggregated and country-specific R^2^ of regression models.

Predictor	eta2_median	eta2_IQR	nld	nor	nzl	per	pol	svk	svn	swe	usa
EDCAT6	6.89	3.32	8.62	8.05	9.76	7.81	3.96	1.91	4.74	9.23	8.19
NUMBOOKS	1.74	1.04	2.07	2.6	2.53	0.87	1.48	2.18	0.54	3.02	1.31
READHOME_Q	1.31	0.87	1.66	1.64	2.15	0.52	0.26	0.38	0.61	1.89	0.76
NUMHOME_Q	1.2	0.9	1.28	1.62	1.75	0.33	1.53	1.77	0.58	1.22	1.56
AGEG5LFS	1.06	1.63	5.1	5.1	1.87	0.58	0.36	0.6	0.73	5.07	0.8
HEALTH	0.73	0.82	0.43	0.82	0.73	1.11	0.98	0.22	1.08	0.28	2.27
LEARNATWORK_Q	0.65	0.68	1.6	0.4	1.2	0.23	0.34	0.63	0.51	2.05	2.05
NUMWORK_Q	0.45	0.34	0.45	1.38	0.97	0.13	0.35	0.12	0.32	1.98	0.82
READYTOLEARN_Q	0.4	0.43	1.02	0.23	0.86	1.15	0.36	0.87	0.44	0.33	0.34
READWORK_Q	0.34	0.21	0.36	0.48	0.4	0.15	0.35	0.31	0.37	0.45	0.33
EDWORK	0.3	0.39	0.57	0.16	0.08	0.3	0.29	0.25	1.03	0.09	0.16
MOTHER_ED	0.27	0.2	0.1	0.2	0.58	0.26	0.27	0.31	0.47	0.06	0.63
FATHER_ED	0.21	0.14	0.11	0.06	0.23	0.32	0.25	0.15	0.71	0.13	1.25
NFE12	0.08	0.18	0.01	0.1	0.07	0.01	0.14	0.58	0.02	0.03	0.24
GENDER_R	0.06	0.08	0.06	0.15	0.03	0.02	0.08	0.01	0.08	0.04	0.07
IMPAR	0.06	0.09	0.23	0.02	0.1	0.03	0.03	0.13	0.13	0.03	0.03
R2	0.4	0.12	0.46	0.42	0.43	0.34	0.32	0.24	0.35	0.46	0.44

We provided an additional and perhaps more intuitive metric of effect size: i.e., a difference in predicted literacy scores associated with extreme values of a given independent variable (excluding undefined or invalid values coded as 9) divided by the country’s standard deviation in literacy scores. The resulting standardized effects are expressed in units of standard deviation and are comparable across samples. Tables 1–3 report standardized effects for each predictor per country in S1 Section of S1 File. Furthermore, Tables 2–4 below include estimates of standardized effects aggregated across samples (median and IQR).

We begin with the bird’s eye view of predictors of literacy by considering their contributions aggregated over the 33 national samples. Generally, all samples converged on which predictors were more influential than others. Rank-order Spearman correlations between all pairs of countries showed a high degree of agreement (median *ρ* = 0.72), with median correlation coefficients for specific countries ranging from *ρ* = 0.45 (Israel) to *ρ* = 0.85 (France), all *p*s < 0.05.

When aggregated across national samples, effect sizes suggest the following hierarchy of relative importance, from the most to the least important predictor of literacy: formal education; number of books in the childhood household; practicing reading and numeracy at home; age; learning at work; readiness to learn; using numeracy and reading for work, mother’s education; occupational status and father’s education, see Tables 2–4. Other sources of variance were minor. Below we provide details on the magnitude and direction of each effect.

By far, the strongest effect on literacy scores in every sample was associated with formal education (median *η*^2^ = 6.89%, median effect = 0.84 *SD*). A higher level of education consistently came with higher literacy scores, with the biggest step-wise score increase between the lowest (incomplete high school) and the second lowest educational levels (completed high school) in most samples, see plots in S1 File.

The number of books in the parental household (median *η*^2^ = 1.74%, median effect = 0.38 *SD*) was the second strongest predictor of literacy scores: The increase in literacy scores between levels of this factor was roughly linear. This finding confirms the notion that the cultural or scholarly capital of the family provides a long-lasting advantage across the entire adulthood, even when controlled for multiple other factors [61].

Engagement in reading and numeracy activities at home were similarly influential (median *η*^2^ = 1.31% and 1.20%; median effect size = 0.17 and 0.12 *SD*, respectively). A large amount of these intrinsically motivated activities led to higher literacy scores, with somewhat diminishing gains in the upper range of these variables, see Fig 1.

Age (median *η*^2^ = 1.06%; median effect size = -0.23 SD) was a relatively minor contributor to literacy scores when considered across all countries. Younger individuals consistently showed higher literacy scores than their older counterparts. Importantly however, the country-specific effect sizes of age were extremely variable: the IQR of the partial *η*^2^ estimate was about 1.5 times larger (1.63) than the median. We return to this observation below.

Health status also showed a relatively small effect (median *η*^2^ = 0.73%; median effect size = -0.15 SD), with better health associated with higher literacy scores. The effect of Learning at the workplace (median *η*^2^ = 0.65%; median effect size = -0.20) uncovered an interesting finding: the individuals with lower literacy levels were the ones who signalled an increased need to learn on the job. This suggests that workers have a substantial insight into their fit with the information-processing demands of the job: the demands are more challenging for those who are less literate. Readiness to learn (median *η*^2^ = 0.40%; median effect size = 0.09 *SD*) showed that higher levels of intrinsic motivation to pursue knowledge came with higher literacy scores as well. The volume of numeracy activities at work and, to a lesser degree, reading at work (median *η*^2^ = 0.45% and 0.34%) both boosted literacy scores, even if to a more modest degree than numeracy and reading at home (reported above).

Several additional control factors exercised weak to moderate effects on literacy levels. Occupational status (median *η*^2^ = 0.30%; median effect size = 0.06 SD) explained additional variance in literacy. In most countries, the highest literacy scores shown by individuals who pursue both work and education (and thus are likely ready for their distinct demands on information-processing skills), followed by—in decreasing order of mean scores—individuals who work (and are not in training), individuals who are not employed but are in job training, and those not employed and not in training. Finally, a higher level of mother’s and father’s education (median *η*^2^ = 0.27% and 0.21%; median effect size = 0.08 and 0.07 SD, respectively) contributed to explaining small amounts of variance in literacy scores. Remaining predictors (non-formal education pursued in the last 12 months; gender of the respondent; and immigrant status of parents) showed effect sizes below the threshold recommended by [67] as a guideline for a small effect in a multiple regression model (*η*^2^ = 0.20%). We do not discuss these variables further.

### Discussion

The analyses above show that literacy levels are co-determined by a broad range of factors that originate in different psychological, social, and economic domains. As data suggest, the relative contributions of these factors are similar across national samples. For the ease of interpretation, we informally classified all studied factors into three major groups: “experiential” factors, “filters”, and “parental” factors, defined below. The groups are not derived statistically from the available data: rather they represent an attempt to organize individual variables considered above into meaningful groups and evaluate their joint influence on cross-national literacy levels.

The most dominant predictors were the ones tapping into the amount, diversity and quality of practice with information-processing skills that individuals engage in. We informally labeled the group of these predictors “experiential”. These predictors included (in decreasing order of relative importance) formal education, practicing reading and numeracy at home, aging, using reading and numeracy for work, and informal education.

Literacy, whether acquired through relatively short spurts of formal education, in response to workplace demands, or through home-related activities, sees gains when it is used more extensively and losses when it is not. This finding is in line with the long-established role of print exposure in literacy attainment and with the “use it or lose it” account of skill acquisition: practice makes perfect. The effect of age appears to break out from this pattern, however. While linguistic experience and knowledge are known to accumulate with age [50, 53, 68, 69], the effect of age on literacy scores was consistently negative: aging comes with literacy losses. We return to this finding below.

Another strong group of predictors of literacy scores can be informally classified as “filters”. They codify the professional and academic achievements of individuals and their readiness to sustain the achieved level, which—to a large extent—is determined by their literacy. These predictors include formal education, occupational status, and the need to learn at work. Indeed, the very purpose of PIAAC was to evaluate how foundational skills, including literacy, shape educational and labor market opportunities in modern advanced economies. In our opinion, the factors in this group are convenient and strong markers of how fully a person has realized or is capable of realizing their academic and professional potential given their literacy level. In terms of [35], they measure the degree of selectivity imposed by the educational and workplace systems, and perhaps the degree of a person’s fit to the cognitive demands of education and workplace. We note that the dual role that formal education plays—as a source of literacy development and a selective filter—calls for a systematic research effort on separating these two roles and estimating the impact of each to acquisition of information-processing skills [37, 70].

A third group of factors represents the endowment that an individual receives from their family and is labeled “parental”: it includes the number of books in the childhood household and mother’s and father’s education. The positive impact of the family cultural or scholarly capital demonstrably carries over the entire adult life: it is as strong in 25–30 year olds as it is in 61–65 year olds, as shown by non-significant interactions with age. Finally, we mention the independent, relatively minor contributions of factors that do not fall into any group: motivation to learn as a personality trait and self-reported health status.

To quantify contributions of each group of factors to literacy scores, we calculated the proportion of variance that each group accounts for out of the total amount of variance explained by all predictors, or each group’s *η*^2^. Because the assignment of formal education to a specific group is ambiguous and its contribution monumental, for the purposes of this estimation we excluded this factor from both the “experiential” factors or “filters”. Under these conditions, experiential factors accounted for a median of 70.0% of total variance explained in literacy scores by all factors across samples (IQR 8.8%); filters accounted for 22.2% (IQR 6.1%); and parental factors for 50.0% of variance (IQR 10.8%). The summed amount of variance exceeds 100% because some of the variance is shared between experiental factors, filters, and parental factors and is accounted for twice when individual contributions of groups are summed up. What we conclude from this analysis, is that the group of factors related to the amount of practice that people get with literacy-related skills is the strongest determiner of their literacy level. The influence of the family (the “parental” group) is very strong as well, when its component predictors are considered jointly. A non-trivial but the smallest amount of variance is also explained by “filters”, variables indicating one’s fit for an educational or occupational environment.

In sum, the present analysis quantifies, for the first time, the relative contributions of individual predictors and groups of predictors to explaining variance in literacy scores and improves our understanding of what makes a good reader. PIAAC is a dataset geared towards population-level estimates, and statistical guidelines to using PIAAC data specifically warn against making inferences for individuals. Yet we can use the data to describe a hypothetical ideal reader. As expected, this type of reader has acquired and continues to accumulate reading experience over their lifetime from a maximally broad variety of activities (e.g., education, reading for pleasure, reading at work, and engaging in numeracy-related tasks which promote literacy as well). They are also the kind of person whose motivation and ability to enhance literacy is supported by the environment in which they are raised and by the genetic predispositions inherited from parents. Finally, they are a person living in an environment that provides stronger incentives and supports for reaching higher literacy levels, often in the shape of formalized qualifications required for personal, financial or social success.

## Analysis 2: Cross-country differences and age

So far, we analyzed common factors driving literacy distributions across 33 national samples. An equally important question is whether countries show systematic differences in which predictors affect their distributions.

**Fig 2:**
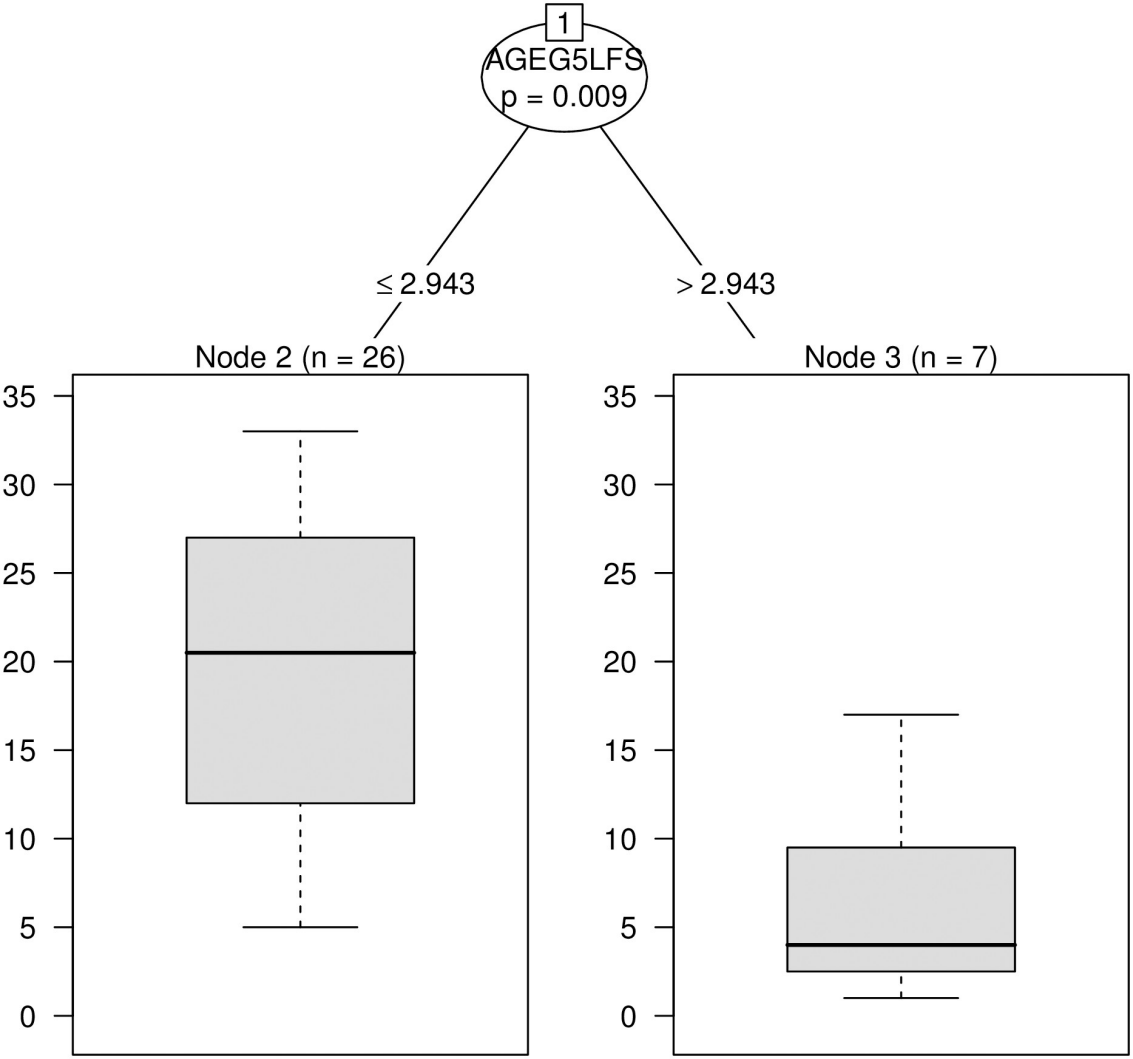
Classification tree of countries based on their literacy score-based ranks.

To this end, a conditional inference tree was fitted to the ranks of national samples based on literacy scores as a dependent variable. We used literacy-based ranks rather than mean literacy scores because of skewness in the distribution of national literacy scores. The predictors used in the conditional inference tree were the same as reported in Tables 2–4. Fig 2 visualizes the output of ctree function in R package party [71].

The resulting conditional regression tree identified age as the only relevant factor for classifying countries into dissimilar groups. Seven countries in which the effect of age was strong (partial *η*^2^ > 2.94, *p* = 0.009) were classified together: Denmark, Finland, Netherlands, Norway, Japan, South Korea and Sweden. These countries have very consistent and very high mean literacy scores (median score = 288, IQR = 9). These countries also rank 1, 2, 3, 4, 6, 14 and 17 on literacy scores in the list of 33 countries. All other countries were grouped together.

### Discussion

The observed pivotal role of age is puzzling, especially given a relatively small effect size of this predictor when considered in aggregation across all countries, Tables 2–4, however see our above discussion of the exceedingly high dispersion of the age effect size. The remainder of this section concentrates on the effect of age. We begin with an overview of the literature on the age effect on literacy skills and then proceed to discuss its components in the light of the present evidence.

#### Components of the age effect

An observable effect of chronological age on any information-processing skill is in fact a conflation of counter-directed effects of multiple components. One component is a lifelong accumulation of experience with the reading skill. This accumulation is evident, for instance, in a continuous increase in vocabulary size with age. Recent lexical decision mega-studies (N > 200,000) in Dutch and English [68, 72] report age as the single most important predictor of vocabulary growth. In native speakers of Dutch, for instance, age has a much stronger positive effect on vocabulary size (*η*^2^ = 17.61%) than the second strongest predictor: education (*η*^2^ = 3.58%). A monotonic age-related increase in vocabulary size has reported in a web-based mega-study (N = 48,000) [73]. A similar growth is expected in those aspects of literacy that remain in active use throughout a person’s lifetime. On the other hand, there may be aspects of the literacy skill that are (mostly) developed during formal education and then remain unused at or outside the workplace and home. It has been argued that these literacy-related skills do not convert into competencies and undergo loss [23, 24], in line with the “use it or lose it” view on skill acquisition [74, 75]. For such skills, age is a metric of loss, which is proportional to a temporal distance from when formal education was completed. Thus, in terms of experience with printed texts, the counter-directed effects of age may co-exist in different domains of the literacy skill. For instance, an overall growth in vocabulary size may take place next to an increasingly lesser proficiency in, say, information search or semantic integration of a text’s content. To our knowledge, little research has been done on disentangling these influences: in childhood [76, 77] or in adulthood [69].

So far, we discussed age only as an index of the amount of practice with language. Yet age is also obviously related to the psychological and physiological processes of aging that accompany maturation [78–81]. In the case of cognitive and especially language-related skills, there is no consensus as to whether or not aging comes with a decline of cognitive functions and especially ones that contribute to language processing [80, 82, 83]. At the same time, there appears to be an agreement that tasks involving lexical knowledge remains fairly unaffected by aging in comparison to tasks recruiting online processes [53, 84, 85]. It is a known fact, however, that the incidence of cognitive disabilities increases with age, even in the 25–65 y.o. span, with some of the disabilities limiting language functioning [86, 87]. Thus, aging as a physiological process may lead to a decrease in the overall literacy levels in the population.

A final component of observable age effect is the impact of a cohort. Forty-year-old participants in 2012 are characterized both by their age (40) and the educational and environmental experiences that differentiate their cohort (born in 1972) from others [24, 88]. In a cross-sectional study like PIAAC, the effect of age is statistically indistinguishable from the cohort effect. To disentangle the two, recent studies [23, 24] use synthetic cohorts, tracking a certain birth cohort through three comparable surveys conducted roughly 9 years apart from one another: see also [88, 89] for alternative approaches. A comparison of the cohorts’ literacy levels across countries and surveys uncovered two major findings. All these studies [23, 24, 88–90] agree that the true effect of aging on literacy, after accounting for the cohort, is negative. That is, younger individuals show higher scores compared to older ones. They disagree however about the magnitude and even the direction of cohort effects at the country level. Barrett and Riddell argue that in several countries (Belgium, Finland, and Netherlands), recent birth cohorts are more literate compared to earlier ones [24]. In these countries, the aging and cohort effects amplify one another. Not only do younger individuals have higher literacy scores than older ones because of the proposed physiological influence of aging, but they also belong to more recent cohorts which have had more opportunities for developing literacy (e.g., better-quality education, or employment or training policies) than subsequent cohorts. Yet in other countries (Canada, USA, Sweden, Norway, and Ireland) more recent cohorts are argued to be less literate than subsequent ones and this effect attenuates the true negative effect of aging. Other reports argue that in every country that they considered more recent cohorts are more literate, over and above aging effects [88].

In the present analysis of cross-sectional data, we are not able to fully break apart the observed effect of age into specific contributions of lifelong practice, skill loss due to under-use, aging, and the cohort effect. It is worth stating that the same methodological limitation is true of any experimental study that compares a group of younger and older participants. However, we provide evidence in favor of some components of the age effect. We discuss these in the following section.

We also note that apparently no other predictor of literacy shows such a conflation of sources of variability as is evidence in the effect of aging. We believe this to be the reason that age was the only predictor differentiating between countries. All other predictors had a similarly strong or weak impact on literacy levels across all countries.

#### Impact of age and its components

The present analyses, as well as multiple prior analyses of PIAAC and similar surveys [56], converge on an observation of a weak *negative* correlation between age and literacy scores, after controlling for other influences. We argue that the correlation is weaker than typically recognized. Earlier reports [23] suggested that the statistical impact of age on literacy is seriously inflated in models that fail to control for other influential predictors of literacy, mainly, education. Indeed, in the present data country-specific regression models that have age as a sole predictor of literacy scores explain between 1% and 20% of variance in the sample’s literacy scores, with a median R^2^ of 6% and an IQR of 8%. If education is included in the hierarchical regression model at step 1 and age at step 2, the amount of unique variance explained by age shrinks to an average of 50% of the original R^2^: with a range of 0–13%, a median of 3% and an IQR of 3%. We argue, however, that even this is an overestimation of the age effect. The reduction is even stronger once a larger set of predictors is considered. In a hierarchical regression model that contains all predictors listed in Analysis 1 as step 1 and age at step 2, R^2^ explained by age further reduces to only 20% of the original R^2^ value: a range of 0–5%, with a median of 1% and a mean of 1.2%. This reduction in the effect size of age is methodologically important: studies that do not control for other influences on literacy scores may inflate the effect of age by a factor of 5. Still the differences in the size of age effect between countries are presently observed even when controlling for a large number of additional factors, which warrants an investigation.

**Fig 3:**
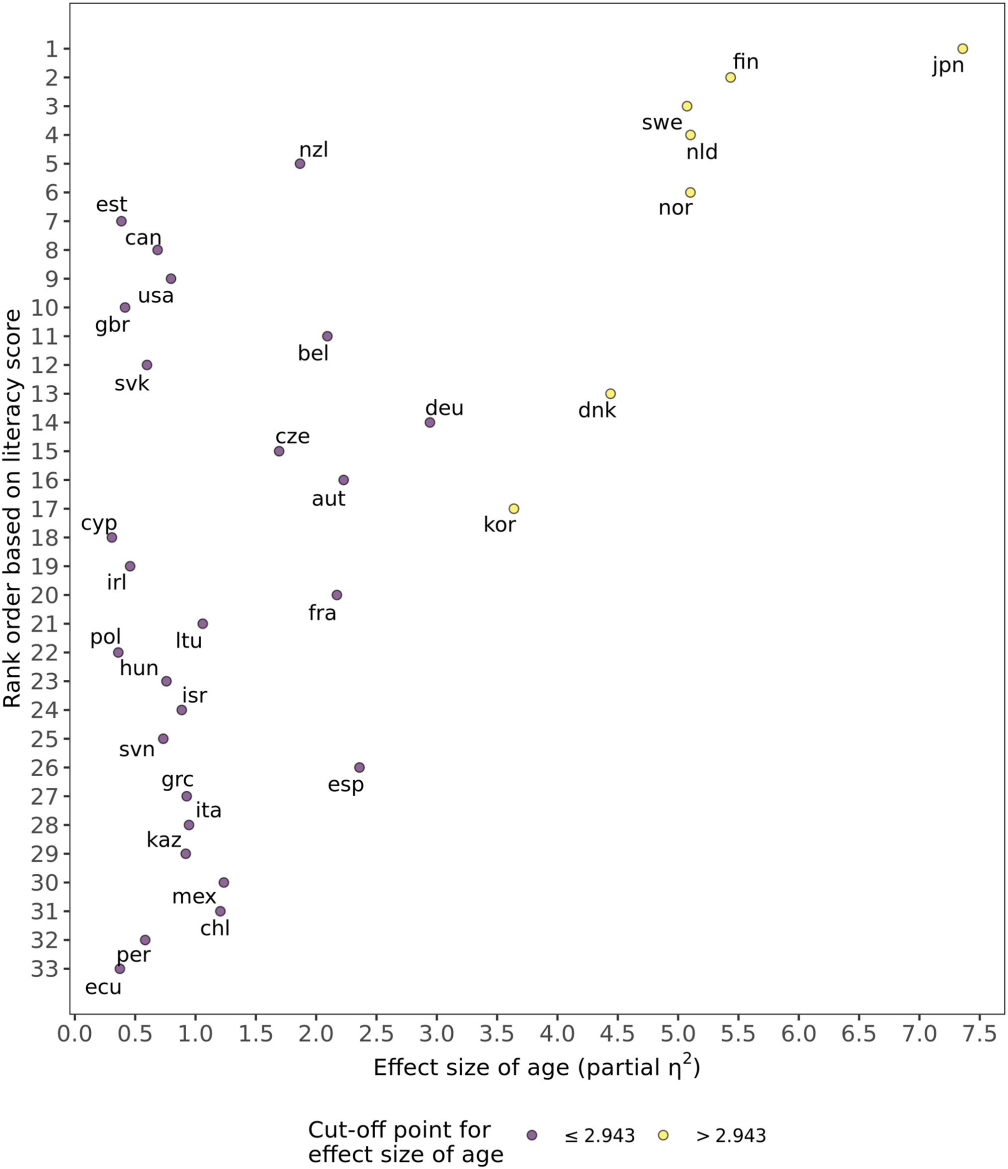
Rank literacy score of the 33 countries relative to the estimated effect size of age. The countries are colored based on the estimated cut-off point of the effect size of age.

To reiterate, age proved to be a pivotal factor in classifying countries based on the effect sizes of predictors of literacy scores. The class of countries with the strongest contribution of age mostly included countries with the highest mean literacy scores. We expand this novel finding by reporting that national samples with the highest average literacy scores show a much stronger effect of age than the remainder of the samples. The Spearman’s rank-order correlation between the effect size of age (partial *η*^2^) and the mean literacy score of the country is *ρ* = 0.49 (*p* < 0.01). The relationship between effect size of age and rank literacy score for a given country is visualized in Fig 3.

There are a few logical possibilities for the observed cross-sample variability in the effect of age. First, it may be that individuals living in the countries with higher literacy scores undergo a more rapid cognitive decline or a larger percentage of them acquires health issues over their lifespan, which limits their opportunities to maintain proficiency in literacy as age advances. This, however, runs counter to the fact that the most literate countries do not fall behind in their average life expectancy compared to other developed countries [91].

A second option may be that factors other than age may be less influential in some samples than others, leaving more variance to be explained by age. This may account for an unequal distribution of the age effect sizes across countries. Yet an analysis of unique variance explained by age with and without being corrected for the influence of other predictors rules out this possibility. Mean literacy scores of countries did not correlate with the amount of variance explained by factors other than age (*p* > 0.1). We conclude that it was not the case that the most-literate countries showed a stronger age effect because other factors (e.g., education, or amount of reading for pleasure or at work) were less influential in those samples than in others.

A final possibility that we consider and find the most plausible is that the fluctuation in the size of the age effect is due to cross-country differences in the magnitude and the direction of the cohort effect. A stronger (compound) effect of age may emerge in those countries where the aging and the cohort effects amplify one another. Older participants in such countries may show lower literacy skills than younger ones both because of accumulating negative consequences of maturation across adulthood and because their cohorts had fewer or lesser-quality opportunities to develop and maintain their literacy skills through education, training, or employment. Our assumption of better educational and employment opportunities for more recent birth cohorts in the countries that we classified together (including Denmark, Finland, Netherlands, Norway, Japan, South Korea and Sweden) agrees with their extremely high standing in the PIAAC literacy tests (ranks 1-5, 14 and 17) and an overall positive correlation between the age effect size and mean literacy score of the country. We note that our data run counter to Barrett and Riddell’s argument [23, 24]. In their analyses of synthetic cohorts from three surveys, some of the countries from the group defined above (Belgium, Finland, and Netherlands) are associated with a positive cohort effect, that is, more recent cohorts are more literate than earlier ones. Yet other countries from that group (Sweden and Norway) are claimed to show a strong cohort effect in the opposite direction, that is, earlier cohorts are more literate than more recent ones. This discrepancy is likely to only be reconciled with longitudinal data at hand, and we discuss this point below.

In the absence of longitudinal data, our above theorizing remains inconclusive. It does, however, lead to falsifiable predictions. We expect that longitudinal research will find (a) a broad variability in the cohort effect sizes, with stronger effects observed in the countries with more literate populations; (b) relatively similar influences on skill coming from aging, and (c) some cross-country variability in age-related effects of accumulation of knowledge and loss due to under-use, commensurate with the countries’ economic, cultural and social affordances for individuals of different age groups.

## General discussion

The overarching goal of this project was to examine the distribution of literacy skills in a large pool of representative national samples and identify the roles of individual predictors and groups of predictors in explaining variance in literacy scores both within and across countries. The motivation was to provide a flourishing field of language research with a comprehensive chart of psychological, social, economic, and cultural factors that contribute to success or failure in meeting the literacy demands of the modern-day technological society. We also sought to establish sources of systematic variability between countries and link them to the overall literacy levels of those countries.

Rich data sources from PIAAC surveys from 33 countries served as the empirical base for this project. These include literacy scores and a massive selection of variables that are comparable across countries. We restricted our analyses to the age range of 25–65 y.o. and to non-immigrant populations who spoke the language of the test as their first language. *Analysis 1* was primarily descriptive and established both the range of relevant predictors of literacy and their relative contributions to explaining variance in literacy scores both in individual countries and in aggregation. Generally, all countries were similar in the hierarchy of predictors of literacy in terms of their relative contributions (median Spearman’s correlation of pairwise country comparisons estimated as partial *η*^2^ effect sizes *ρ* = 0.72). Effect sizes were reported for all predictors and all countries in Tables 2–4. Formal education was by far the strongest predictor of literacy scores in all countries, yet an additional three groups of predictors explained substantial amounts of variance even without taking formal education into account. These were *experiential* predictors that quantify the amount of reading and related activities like numeracy at home and at work (*η*^2^ = 70%); *parental* predictors that tap into the cultural and scholarly capital of the individual’s childhood family (*η*^2^ = 50%); and *filter* predictors that codify academic and professional qualifications and one’s fit to them (*η*^2^ = 22%). We believe that our findings present a fairly complete descriptive picture of what makes people more or less literate, given the available data sources. It is worth mentioning, however, that our regression models only explained about 40% of variance in literacy scores. Thus the task of pinning down non-random variance in individual and country-wide achievements in the literacy domain is far from complete.

An additional finding from Analysis 1 merits a separate discussion. The strong role of *parental factors* in predicting literacy (especially, the number of books in the childhood’s household and mother’s education) reinforces the notion that the problem of inadequate literacy in present-day adults propagates into future generations [69, 92]. A low cultural capital and low educational level in a person, due to their low proficiency in literacy, translates into fewer parental resources offered to their children. To quantify this impact, the joint effect on literacy of the number of books and mother’s education is such that the lowest and the highest levels of parental capital lead to a difference of 0.4 *SD* in literacy scores. This is the same magnitude of difference in mean literacy scores of an average respondent with a completed high school diploma and one with a completed Master’s/PhD degree. Thus, success or failure of a nation to procure advancement in the literacy level of a current workforce have a noticeable impact both on the present and future society as well as economy.

*Analysis 2* examined whether variability in the country-specific distributions of literacy skills is systematic and whether it can be explained by one or more individual predictors of literacy. The systematic nature of variability was revealed in the classification analysis, which grouped together countries with the highest and very consistent literacy scores (Denmark, Finland, Netherlands, Norway, Japan, South Korea and Sweden) versus countries with lower and more variable literacy scores, see Fig 2. That is, countries that are similar to one another in their level of literacy tend to show a similar hierarchy of relative contributions of individual predictors.

Furthermore, Analysis 2 demonstrated for the first time that the cross-country variability in literacy achievements is at least partly related to fluctuations in the effect of age. Thus, age was a primary factor that determined classification of countries by their literacy score-based ranks, described above (Fig 2). This suggests that the effect of age on literacy determined, to a large degree, whether countries were similar to each other in terms of the relative importance of individual predictors. This was true even though our regression models with large number of predictors gave a more realistic picture of the minor amount of variance that age explained, as compared to prior research where the effect of age could be constrained by only a few predictors. An additional finding was that countries with higher mean literacy scores showed a stronger effect of age on literacy scores: *ρ* = 0.49, *p* < 0.01. That is, the better a country’s population performed in the literacy test, the more decline was observed in literacy scores as a function of age.

To interpret this finding, we reviewed and tested several components of the age effect that the literature proposed. Briefly, the effect of age is a conflation of aging (maturational changes in one’s cognitive and physiological functioning over time) and accumulation of linguistic and other knowledge and experience over the lifetime, and possibly other factors (see references in Analysis 2). Thus, with age a person is more likely to experience health problems affecting cognitive performance and potentially level of literacy; this person is, however, likely to also gain experience in at least some literacy-related domains (most notably, in vocabulary growth). Moreover, an age effect is confounded with a cohort effect and thus a decrease in literacy scores over time may have a true age component but also be a function of differences in the quality and quantity of education and workplace demands across different cohorts.

The present cross-sectional data did not allow us to disentangle all of the components and account for which of them drove the observed impact of age. Yet our analyses allowed us to rule out some possibilities. Thus, we did not find independent evidence for the possibility that most literate countries are also the ones where the maturational effects of aging (e.g., the rate of cognitive impairments) are stronger or more common than in less literate countries. The most likely culprit for the pivotal role of age in explaining variability between countries is the cohort effect. We hypothesize that the countries which show higher literacy scores and, relatedly, a stronger negative effect of age on literacy are those countries where the cohort effect amplifies and is co-directed with the age effect. In other words, these are the countries where older individuals have poorer performance compared to other countries primarily because the more recent cohorts in these countries are trained consistently better than the less recent ones. Under our hypothesis, what comes out as a fluctuation in the size of the age effect is in fact a difference in the evolution of the educational, social and economic systems, which led to a more substantial incremental improvement in literacy skills of more recent cohorts and to greater overall literacy levels in some countries but less so in others. We note that our findings run counter to the those of who used multiple surveys to create synthetic cohorts and observed a cohort effect that amplifies the age effect in some of the most literate countries but attenuates the age effect in others (see above for detail) [23, 24]. Longitudinal data will be required to validate our hypothesis or Barrett and Riddell’s findings.

### Future directions

We supplement our findings with a few methodological recommendations and pointers to future directions of research. First, one of the strongest individual predictors of literacy across countries was the number of books at one’s childhood’s household. The PIAAC entry for the question reads: “About how many books were there in your home when you were 16 years old? Do not include magazines, newspapers or schoolbooks. To give an estimation, one meter of shelving is about 40 books.” and has the following possible answers: 10 books or less, 11–25, 26–100, 101–200; 201–500; more than 500 books; and don’t know. Given the brevity of administering this question, we suggest routinely collecting this information in all reading experiments on adults and including its outcome as a co-variate of reading performance.

Second, despite the wealth of literature supporting its importance for literacy development (see references above), informal education proved to have negligible impact on literacy skill. We speculate that this weak effect is either because the PIAAC questionnaire only asks whether this education was pursued in the last 12 months and misses its long-term impact, or the impact of non-formal education is absorbed by other, more influential variables (e.g., reading at home or readiness to learn). This suggests that future surveys may consider a scrutiny of non-formal education on a longer timescale.

Third, the cross-country variability in the size of the age effect suggests that results of experimental studies on aging effects on reading, literacy, numeracy and related skills in adults are not readily comparable across countries. For instance, while Canada, UK and USA demonstrate a low impact of age on literacy, several Nordic countries as well as Japan show a markedly stronger impact. In this setup, a typical experiment that compares young adults (20–25 y.o.) to older adults (65+ y.o.) in a language related task would uncover stronger effects in the Finnish or Dutch sample than in the Canadian or American one. This age × country interaction would likely be spurious and would reflect differences in the cohort effect that cannot be accounted for in a cross-sectional study. In addition, to correctly estimate the effect of age both in within- and between-countries studies, it is important to include other relevant predictors of literacy and reading performance (e.g., education, measures of cultural capital, reading habits, etc). In the absence of such predictors, the effect of age tends to be greatly inflated. As shown above, the effect of age on literacy reduced to 50% of the original effect size when controlling for education and to as little as 20% of the original when controlling for other predictors.

Fourth, the present data point to a consistent negative effect of age on literacy. This effect runs counter to the well-established positive effect of age on vocabulary growth [53, 68, 76] and grammatical ability [73] in recent mega-studies. Being cross-sectional, these mega-studies are expected to demonstrate the same conflation of the aging and cohort effects as the one hypothesized for PIAAC, thus study design cannot account for the discrepancy. We speculate that different aspects of the literacy skill are affected by age to a different degree and perhaps even in the opposite ways. A fruitful future direction would be to find out which specific skills are on the rise (e.g., vocabulary growth) and which are in decline as age progresses.

We also note as a limitation that immigrant groups were excluded from the current cross-national study, because of the well-known rampant variability in the number and social, educational, and demographic characteristics of respective populations in different countries (e.g., immigrants only account for 0.02% of the Japanese PIAAC sample, while immigrants and individuals with home language other than one of official languages constitute 29% of the Canadian sample) [93–96]. We believe that a cross-national comparison of literacy skills among immigrants is an important topic to pursue in future research.

## Supporting information

S1 File (PDF)
