# Supplementary material for: Predictors of literacy in adulthood: Evidence from 33 countries
Source: PLoS One. 2021 Mar 11;16(3):e0243763. doi: 10.1371/journal.pone.0243763 (PMC7951848; doi:10.1371/journal.pone.0243763)
Supplement: S1 File — (PDF) [file pone.0243763.s001.pdf]

Supplementary materials for Predictors of literacy in adulthood: Evidence from 33 countries

Aki-Juhani Kyröläinen[1,2◕], Victor Kuperman[1◕]

1 Department of Linguistics and Languages, McMaster University, Hamilton, Ontario, Canada
2 Department of Applied Linguistics, Brock University, St. Catharines, Ontario, Canada

◕These authors contributed equally to this work.
¶Membership list can be found in the Acknowledgments section.
* akkyro@gmail.com

1 S1: Standardized effect size

The standardized effects of predictors of literacy were aggregated for each country and are presented in Table 1, 2 and 3. The countries are given in alphabetical order.

Fig 1. Partial effects of predictors on literacy scores in the Canadian sample. Error bars represent the 95% confidence interval. Value "9" stands for missing or undefined responses excluding the predictor age (AGEG5LFS).

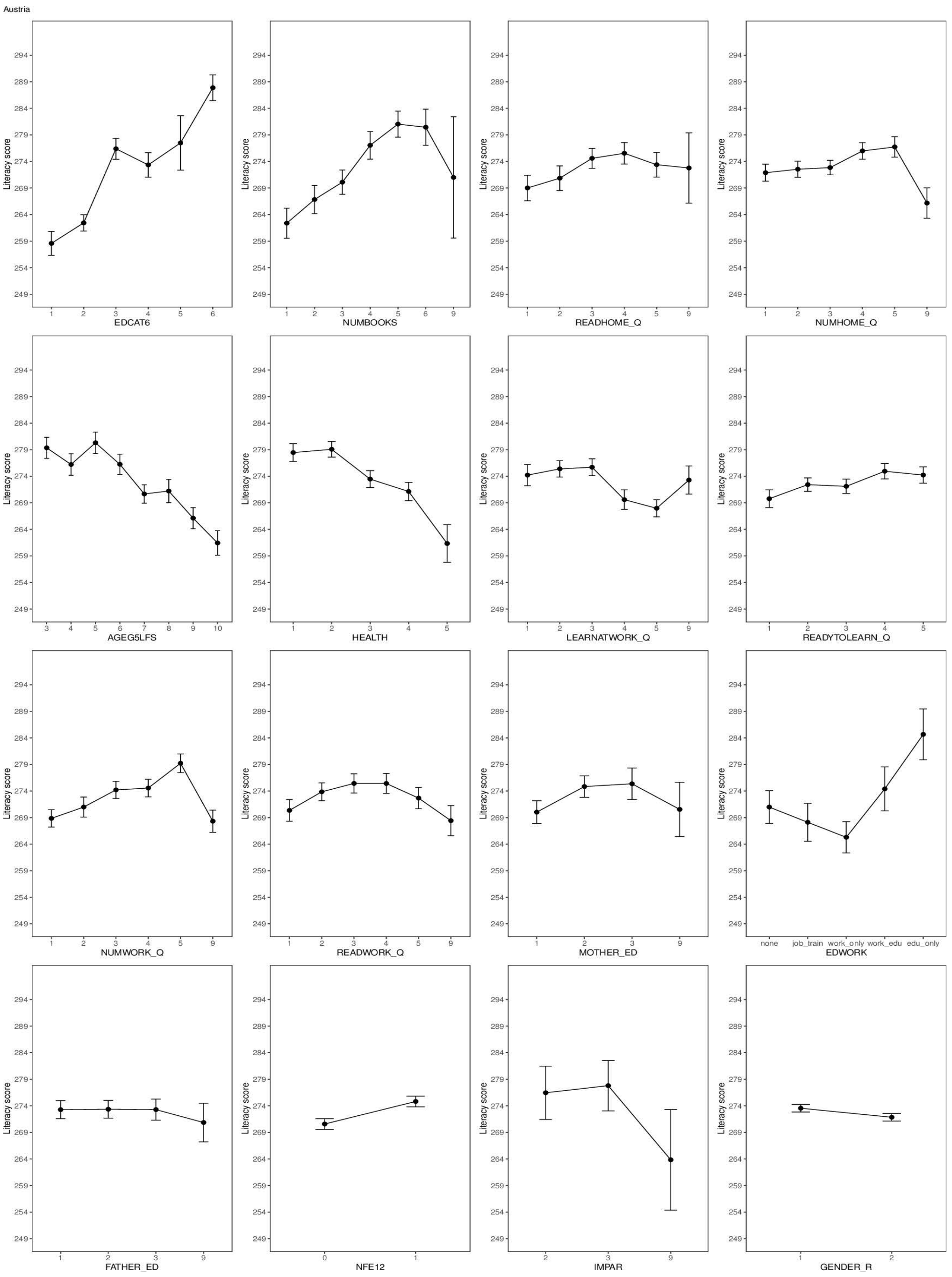

2 S2: Partial effects

In this section, the estimated partial effects are visualized separately for each country from Fig 2 to Fig 33. The countries are presented in alphabetical order. Please note that due to a large variation in the literacy scores across the countries (for example compare the range of the literacy scores between Japan and Ecuador), the estimated partial effects are visualized on the same scale only for a given country.

Fig 2. Partial effects of predictors on literacy scores in the Austrian sample. Error bars represent the 95% confidence interval. Value “9” stands for missing or undefined responses excluding the predictor age (AGEG5LFS).

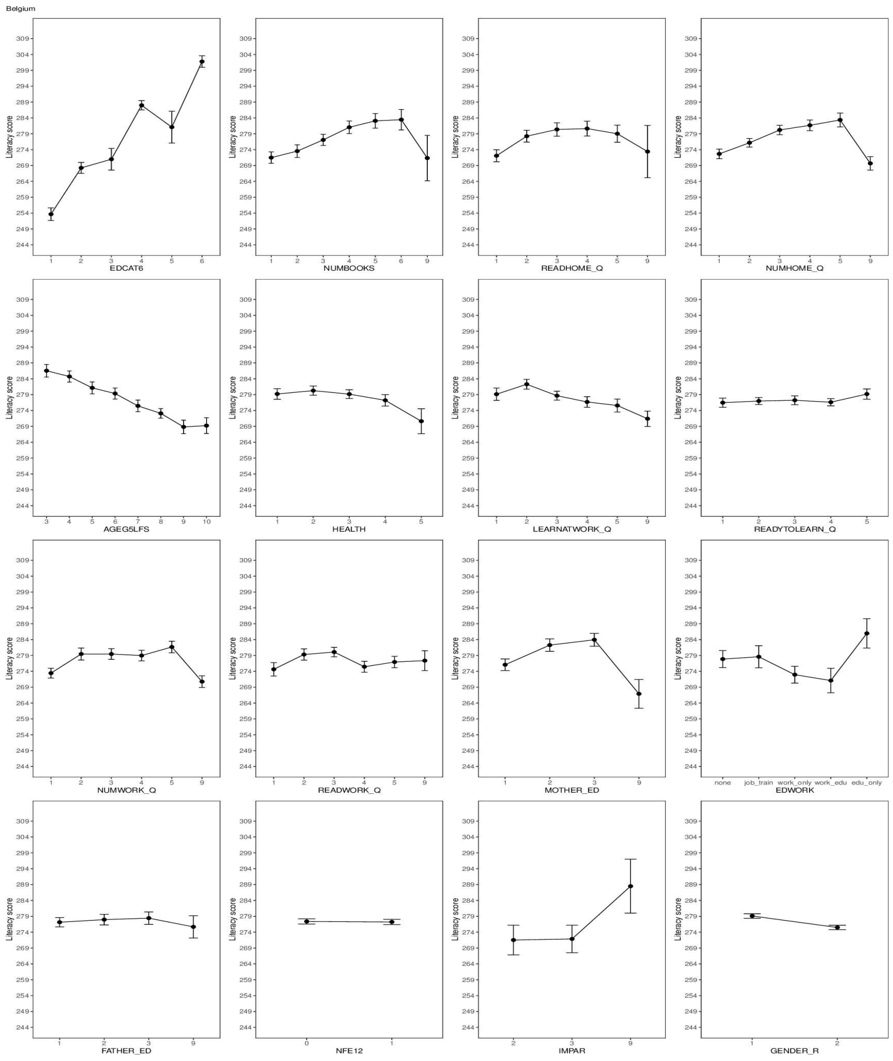

Fig 3. Partial effects of predictors on literacy scores in the Belgian sample. Error bars represent the 95% confidence interval. Value "9" stands for missing or undefined responses excluding the predictor age (AGEG5LFS).

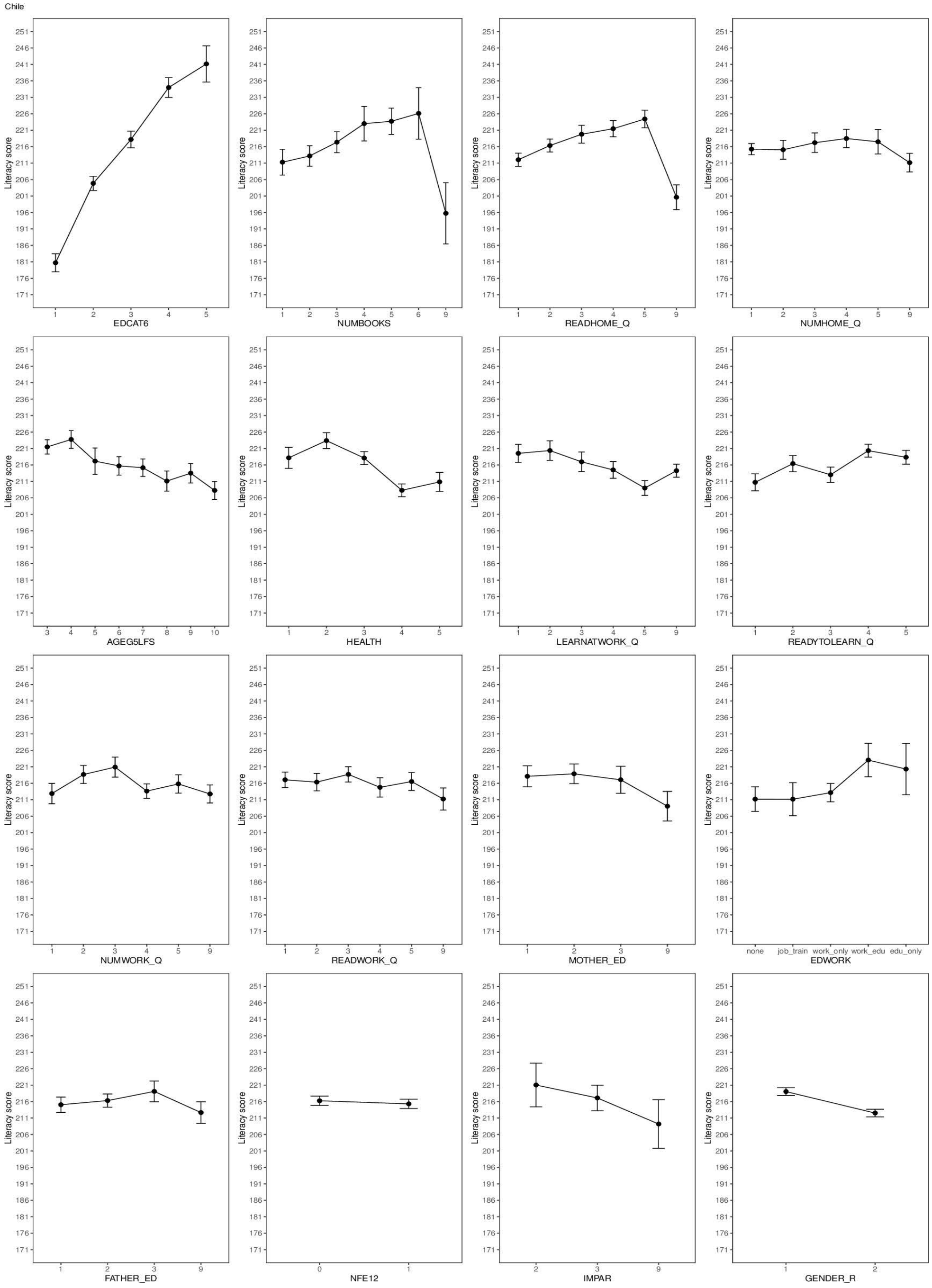

Table 1. Standardized effects of predictors of literacy aggregated by country (from Austria (aut) to Estonia (est)).

	median	IQR	aut	bel	can	chl	cyp	cze	deu	dnk	ecu	esp	est
EDCAT6	0.8	0.22	0.79	1.06	1.17	1.27	1.22	0.86	0.9	0.8	0.36	0.79	0.78
NUMBOOKS	0.35	0.14	0.43	0.33	0.29	0.33	0.08	0.28	0.45	0.36	0.48	0.41	0.49
READHOME_Q	0.19	0.16	0.11	0.16	0.27	0.14	0.11	0.19	0.26	0.45	0.23	0.2	0.08
NUMHOME_Q	0.22	0.09	0.14	0.22	0.28	0.14	0.11	0.37	0.22	0.3	0.1	0.27	0.35
AGEG5LFS	-0.16	0.21	-0.42	-0.38	-0.12	-0.16	0	-0.12	-0.22	-0.53	0.09	-0.23	-0.15
HEALTH	-0.14	0.15	-0.16	-0.14	-0.18	-0.07	-0.17	-0.36	-0.28	-0.36	0.11	-0.38	-0.09
LEARNATWORK_Q	-0.18	0.12	-0.16	-0.03	-0.24	-0.2	-0.07	-0.04	-0.18	-0.17	0.03	-0.04	-0.19
READYTOLEARN_Q	0.08	0.1	0.1	0.07	0.17	0.12	-0.09	0.02	0.12	0.08	0.23	0.01	0.03
NUMWORK_Q	0.12	0.08	0.27	0.2	0.12	0.06	-0.04	0.14	0.14	0.16	0.14	0.11	0.16
READWORK_Q	-0.02	0.09	0.03	0.07	-0.02	-0.09	-0.24	-0.03	-0.06	0.05	-0.12	0.07	-0.14
MOTHER_ED	0.08	0.09	0.15	0.19	0.11	0.02	-0.08	0.06	0.02	0.08	0.3	-0.07	0.1
EDWORK	0.08	0.18	0.16	0.01	-0.01	0.19	0.21	0.17	0.21	-0.1	0.14	0.25	0.31
FATHER_ED	0.08	0.07	-0.09	0.02	0.05	0.01	0.06	0.17	0.06	0.09	0.03	0.09	0.01
NFE12	0	0.05	0	-0.01	0.11	0	0	0.07	0	0.02	0.11	0	0.06
IMPAR	0.01	0.29	0.15	0.01	-0.06	-0.18	-0.77	-0.05	0.01	0.45	0.24	0.02	0.32
GENDER_R	-0.02	0.05	-0.02	-0.08	-0.02	-0.06	0.02	-0.03	0.02	0.01	0.1	-0.06	-0.01

Table 2. Standardized effects of predictors of literacy aggregated by country (from Finland (fin) to South Korea (kor)).

	median	IQR	fin	fra	gbr	grc	hun	irl	isr	ita	jpn	kaz	kor
EDCAT6	0.8	0.22	0.77	0.55	0.75	0.64	0.93	0.99	0.72	0.49	1.08	0.49	1.15
NUMBOOKS	0.35	0.14	0.49	0.35	0.3	0.52	0.39	0.31	0.04	0.5	0.46	0.35	0.17
READHOME_Q	0.19	0.16	0.46	0.11	0.32	0.17	0.19	0.26	0.02	0.1	0.14	-0.05	0.14
NUMHOME_Q	0.22	0.09	0.24	0.24	0.19	0.19	0.23	0.25	0.17	0.02	0.16	0.09	0.06
AGEG5LFS	-0.16	0.21	-0.57	-0.37	-0.05	0.31	-0.1	-0.15	-0.03	-0.16	-0.48	0.2	-0.43
HEALTH	-0.14	0.15	-0.2	-0.09	-0.2	0.03	-0.19	-0.15	-0.47	0.02	0.06	0	0.01
LEARNATWORK_Q	-0.18	0.12	-0.21	-0.02	-0.27	-0.3	-0.31	-0.05	-0.21	-0.33	-0.18	-0.42	-0.18
READYTOLEARN_Q	0.08	0.1	0.04	0.13	0.05	0.01	-0.02	0.09	0.35	0.03	0.03	-0.01	-0.01
NUMWORK_Q	0.12	0.08	0.19	0.1	0.15	-0.01	0.14	0.03	0.04	0.23	0.09	0.03	0.15
READWORK_Q	-0.02	0.09	0.08	0.04	0.08	0.06	-0.04	-0.15	-0.03	0.04	-0.09	-0.12	-0.15
MOTHER_ED	0.08	0.09	0.1	0.06	0.16	0.19	0.17	0.07	0.24	0.06	0.01	0.16	-0.04
EDWORK	0.08	0.18	0.07	0.04	0.06	-0.09	0.13	0.08	-0.01	0.15	-0.36	0.7	0.21
FATHER_ED	0.08	0.07	0.08	0.12	0.1	0.07	0.1	0.1	0.16	0.08	0.04	-0.06	0.09
NFE12	0	0.05	0	0	0	0	0.12	0	0.06	0	0	0	0.09
IMPAR	0.01	0.29	0.14	0.13	0.18	-0.07	-0.07	-0.15	-0.09	0.64	-0.17	-0.33	-0.01
GENDER_R	-0.02	0.05	0.01	-0.02	-0.04	0.11	0.06	-0.12	-0.03	0.03	-0.03	0.02	-0.09

Table 3. Standardized effects of predictors of literacy aggregated by country (from Lithuania (ltu) to the United States (usa)).

	median	IQR	ltu	mex	nld	nor	nzl	per	pol	svk	svn	swe	usa
EDCAT6	0.8	0.22	0.76	0.63	0.88	0.74	0.87	1.05	0.7	0.7	0.82	0.8	1.05
NUMBOOKS	0.35	0.14	0.12	0.66	0.24	0.48	0.37	0.3	0.42	0.32	0.14	0.42	0.26
READHOME_Q	0.19	0.16	0.18	0.22	0.39	0.71	0.45	0.05	0.07	-0.08	0.19	0.31	0.2
NUMHOME_Q	0.22	0.09	0.21	0.13	0.25	0.26	0.25	0.06	0.27	0.21	0.22	0.28	0.41
AGEG5LFS	-0.16	0.21	-0.33	-0.26	-0.38	-0.55	-0.16	-0.14	-0.01	-0.09	-0.2	-0.3	-0.18
HEALTH	-0.14	0.15	-0.17	-0.05	-0.05	-0.21	-0.11	-0.04	-0.3	0.07	-0.06	0.1	-0.25
LEARNATWORK_Q	-0.18	0.12	-0.29	-0.06	-0.23	-0.18	-0.21	-0.16	-0.18	0	-0.07	-0.4	-0.32
READYTOLEARN_Q	0.08	0.1	-0.11	0.21	0.12	0.14	0.25	0.28	0.07	0.1	0.12	0.16	-0.01
NUMWORK_Q	0.12	0.08	0.07	0.11	0.11	0.23	0.17	0.08	0.16	0.03	0.11	0.36	0.04
READWORK_Q	-0.02	0.09	0.05	-0.07	0.13	0.01	-0.03	-0.02	0.08	0	0.15	-0.03	0.07
MOTHER_ED	0.08	0.09	0.01	0.01	0.03	0.11	0.07	0.03	0.24	0.21	0.2	0.06	0.19
EDWORK	0.08	0.18	0.07	0.09	-0.08	-0.19	-0.1	0	0.17	-0.02	0.16	0	0.08
FATHER_ED	0.08	0.07	0.09	0.12	-0.01	0.03	0.08	0.17	0.1	0.09	0.17	0.05	0.24
NFE12	0	0.05	0	0	-0.03	0	0.06	-0.05	0	0.16	0.02	0.02	0
IMPAR	0.01	0.29	-0.26	-0.01	0.09	0.02	0.05	0.98	-0.23	-0.06	-0.02	0.16	0.17
GENDER_R	-0.02	0.05	0.01	0.08	-0.03	-0.08	0	0.01	0.05	0.02	-0.06	-0.04	-0.03

Fig 4. Partial effects of predictors on literacy scores in the Chilean sample. Error bars represent the 95% confidence interval. Value "9" stands for missing or undefined responses excluding the predictor age (AGEG5LFS).

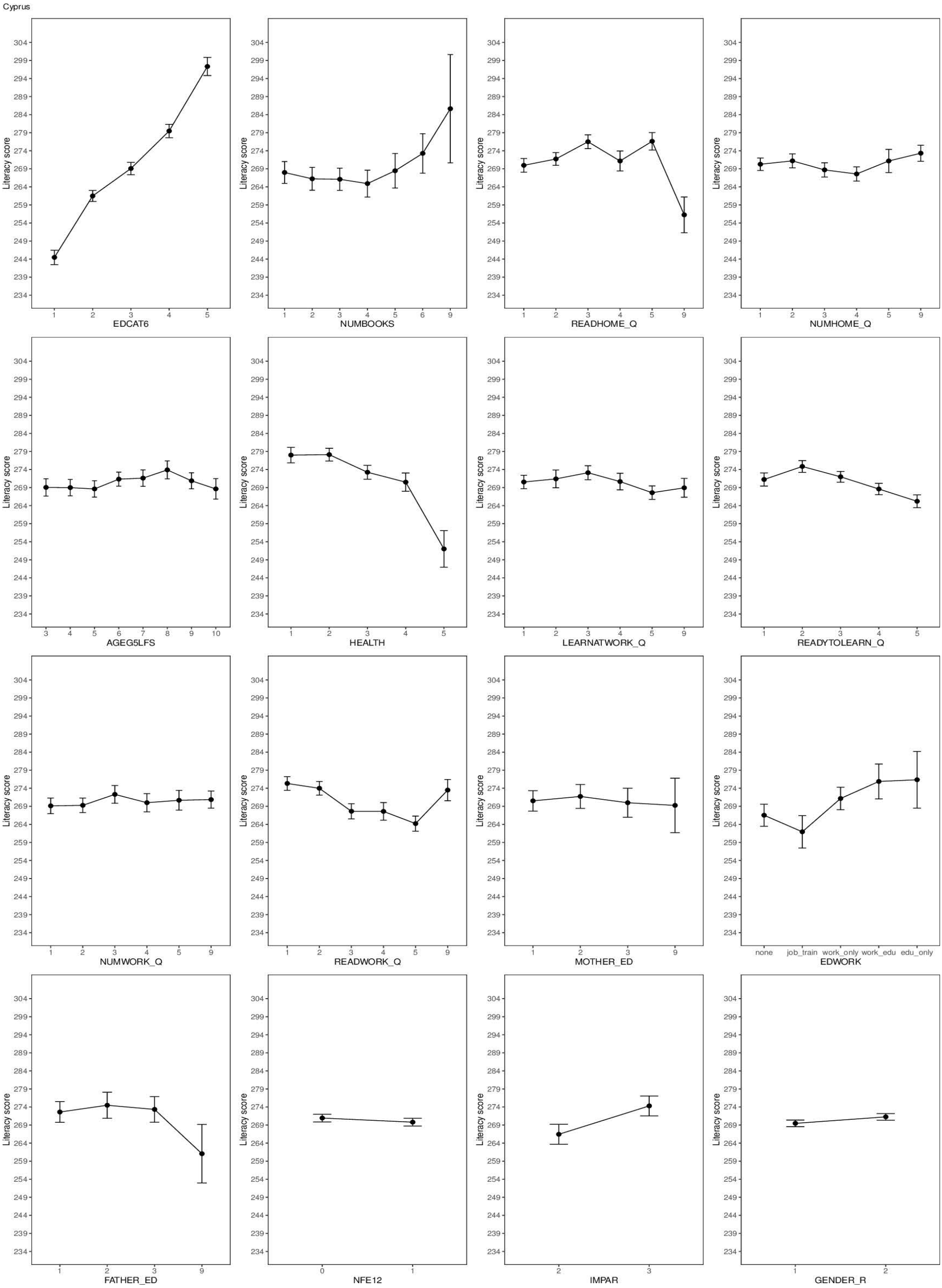

Fig 5. Partial effects of predictors on literacy scores in the Cypriot sample. Error bars represent the 95% confidence interval. Value "9" stands for missing or undefined responses excluding the predictor age (AGEG5LFS).

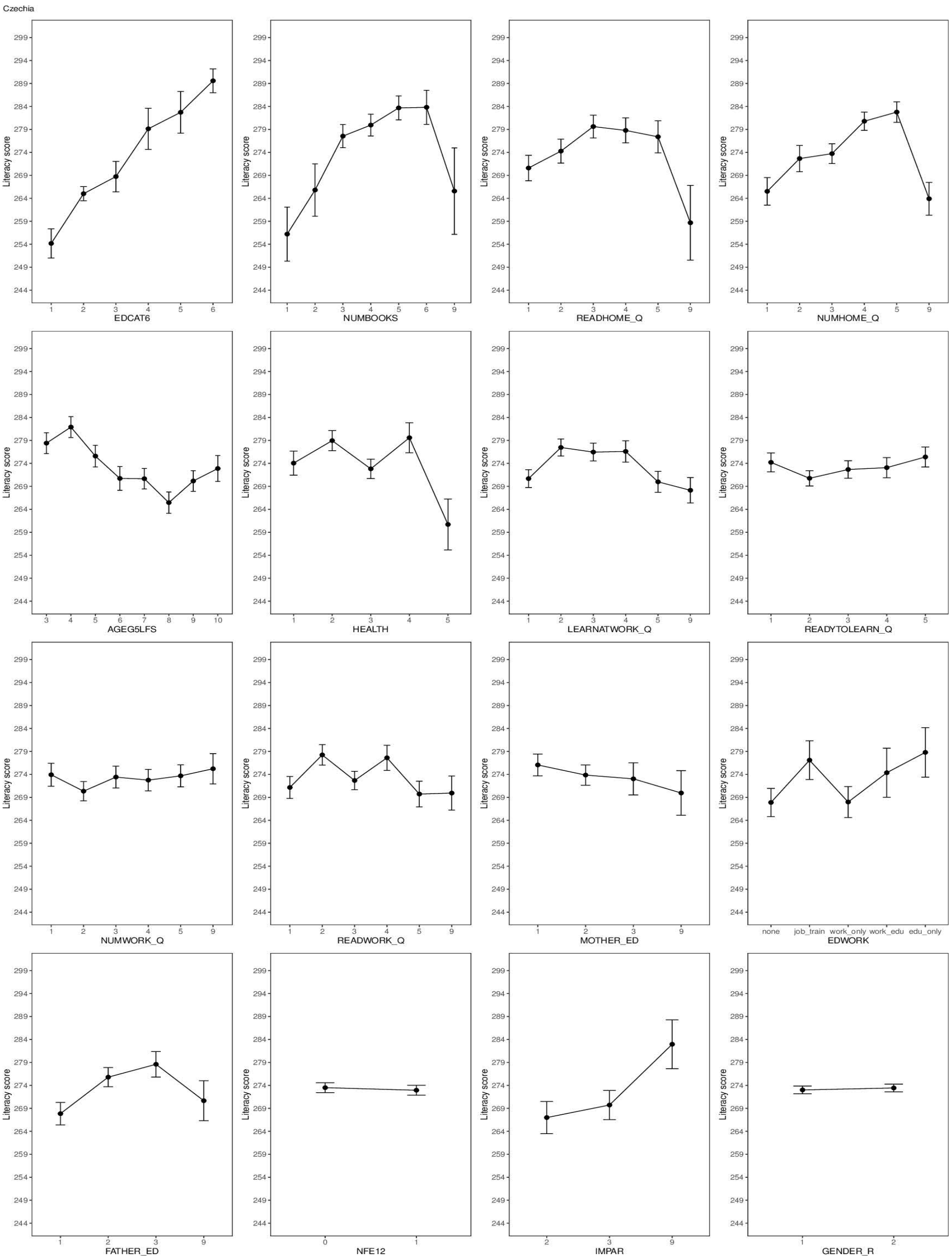

Fig 6. Partial effects of predictors on literacy scores in the Czech sample. Error bars represent the 95% confidence interval. Value "9" stands for missing or undefined responses excluding the predictor age (AGEG5LFS).

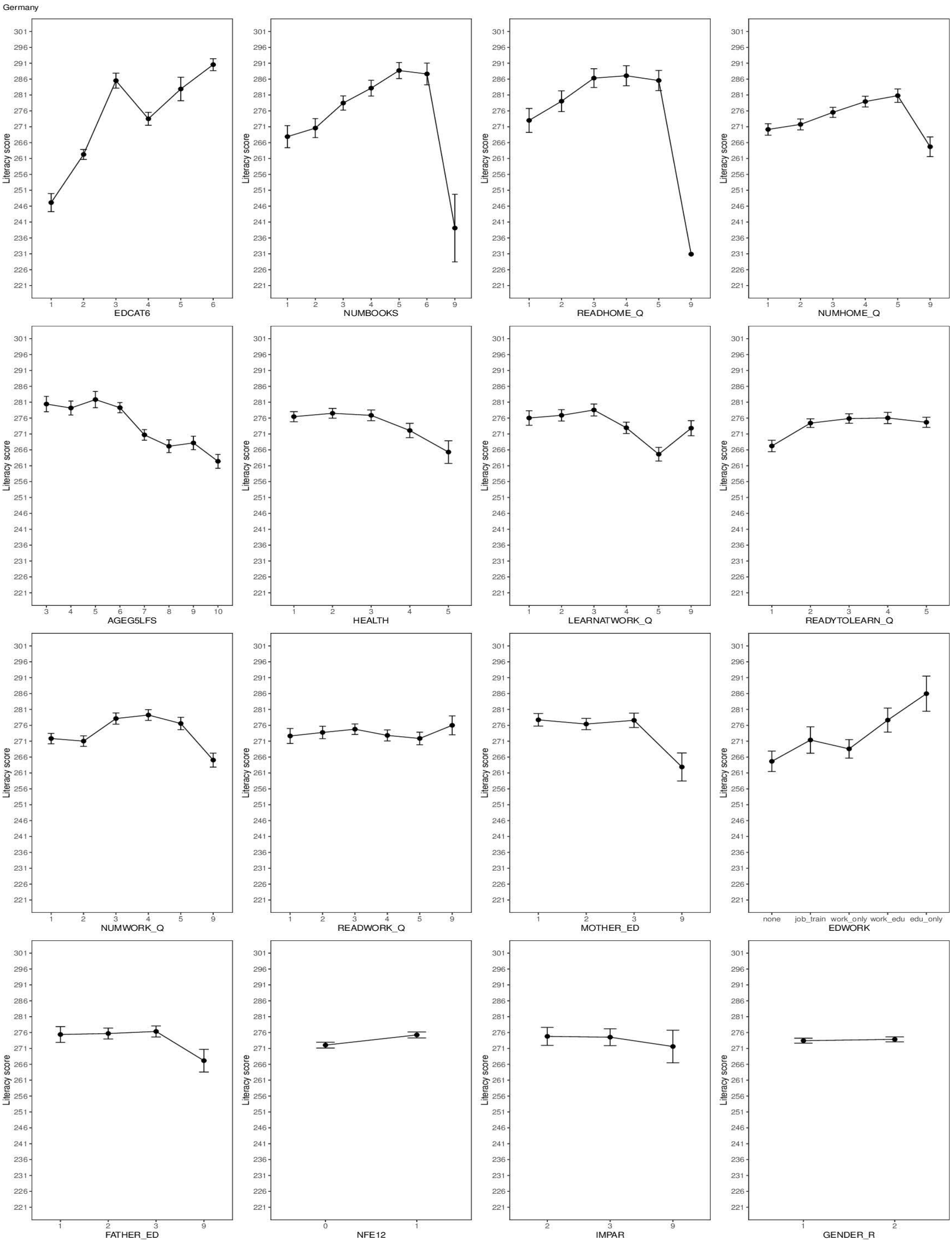

Fig 7. Partial effects of predictors on literacy scores in the German sample. **Error bars represent the 95% confidence interval. Value “9” stands for missing or undefined responses excluding the predictor age (AGEG5LFS). Due to increased uncertainty, some of the CIs were clipped.**

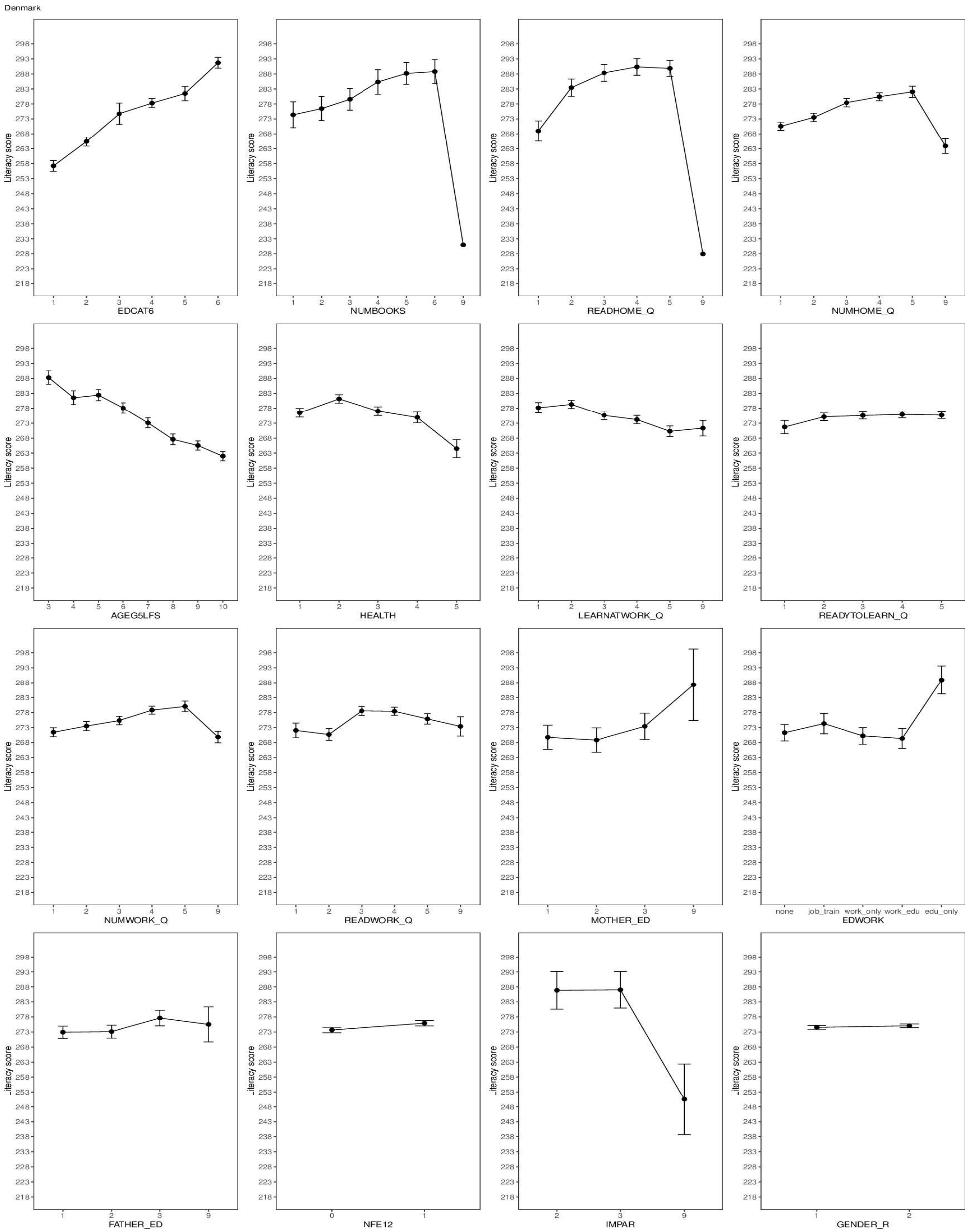

Fig 8. Partial effects of predictors on literacy scores in the Danish sample. **Error bars represent the 95% confidence interval. Value "9" stands for missing or undefined responses excluding the predictor age (AGEG5LFS). Due to increased uncertainty, some of the CIs were clipped.**

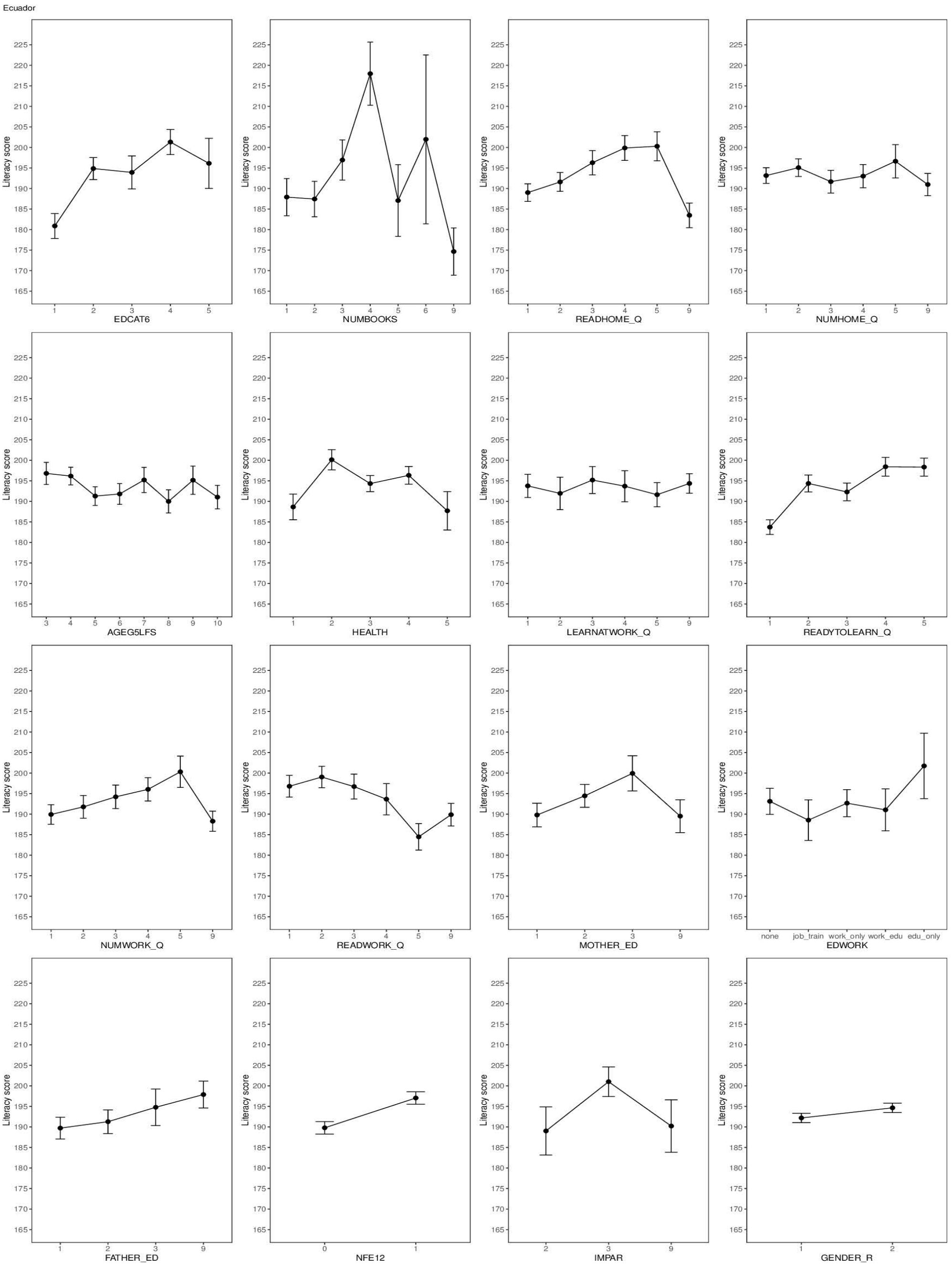

Fig 9. Partial effects of predictors on literacy scores in the Ecuadorean sample. Error bars represent the 95% confidence interval. Value "9" stands for missing or undefined responses excluding the predictor age (AGEG5LFS).

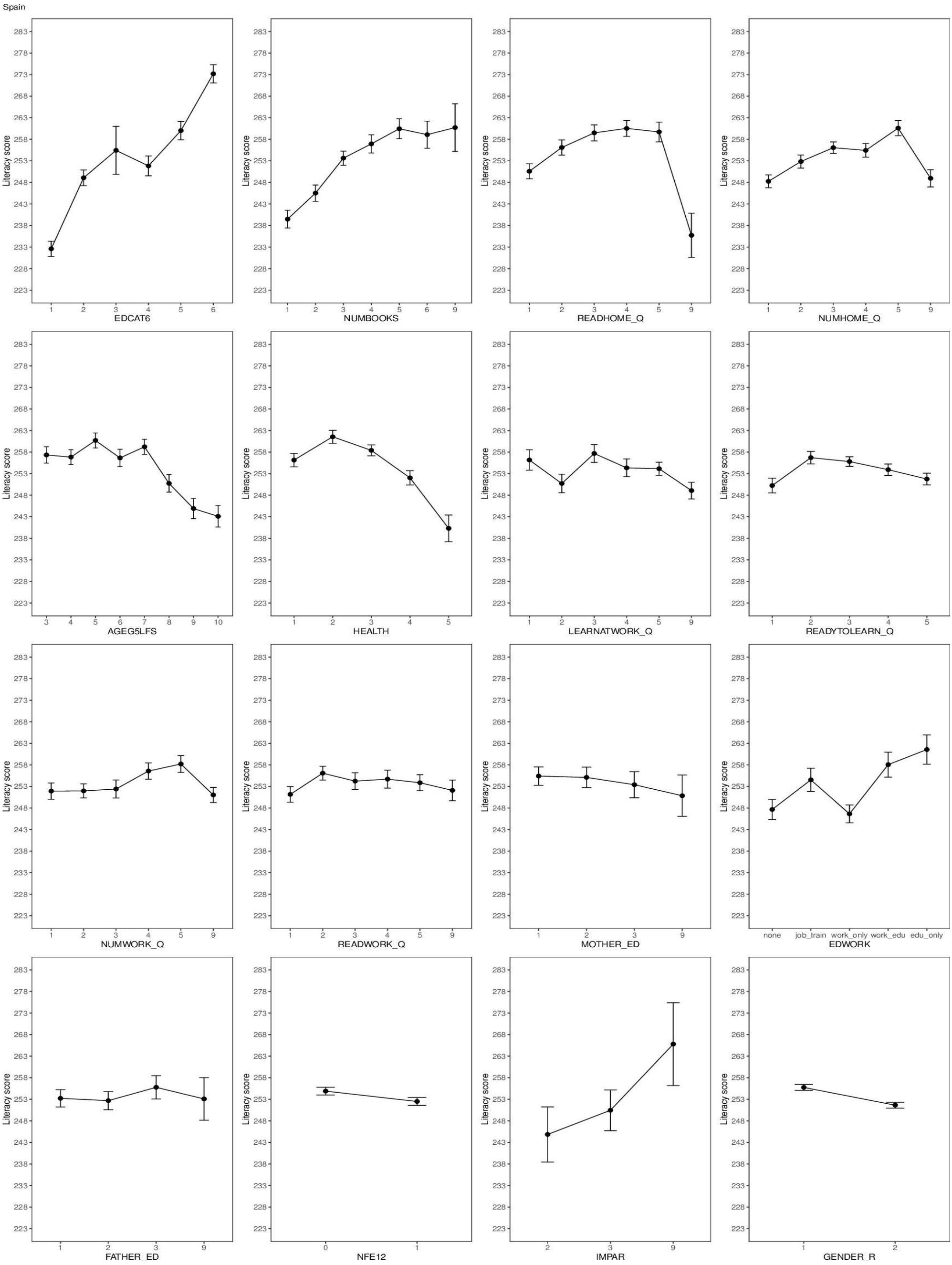

Fig 10. Partial effects of predictors on literacy scores in the Spanish sample. Error bars represent the 95% confidence interval. Value "9" stands for missing or undefined responses excluding the predictor age (AGEG5LFS).

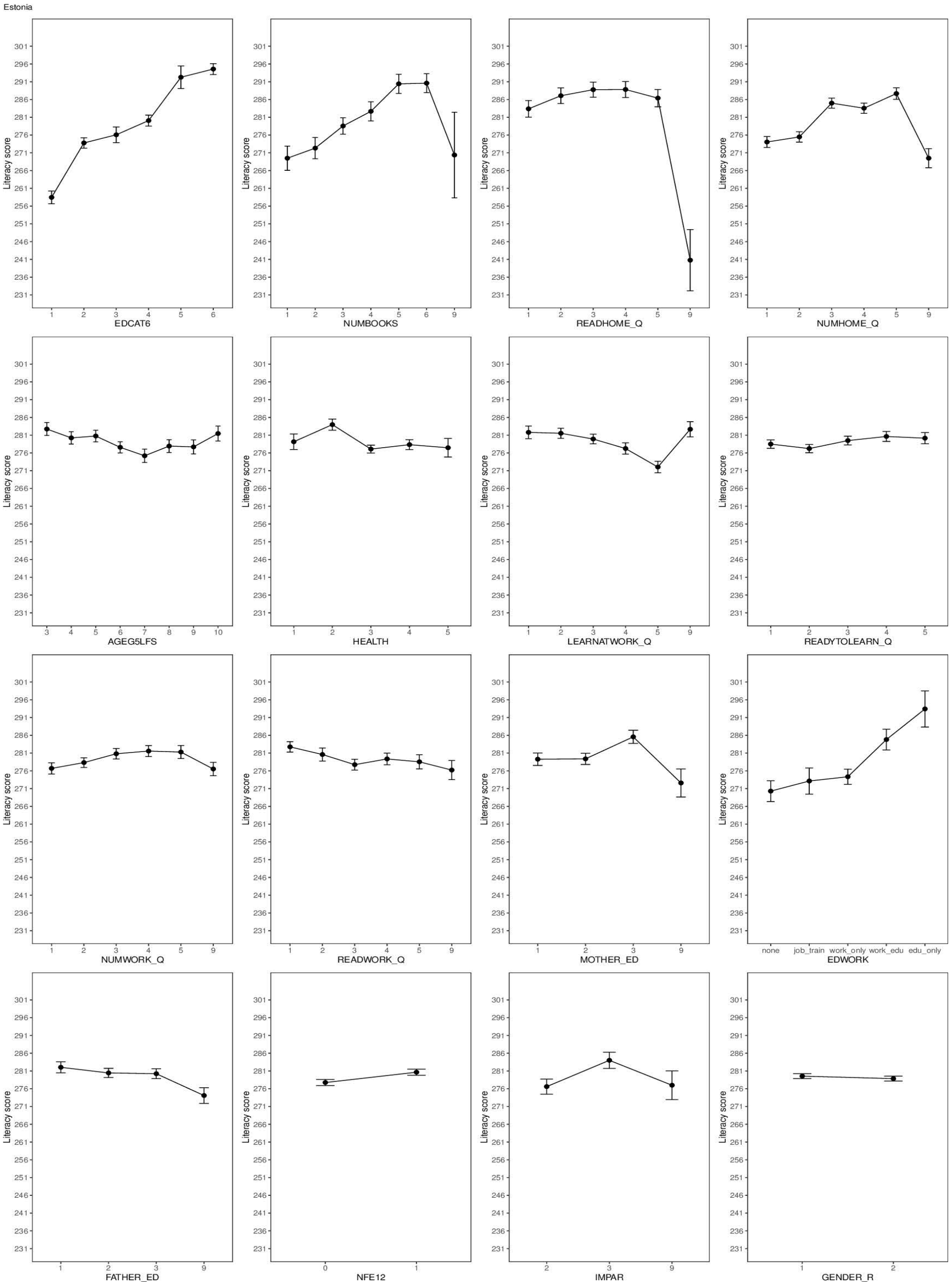

Fig 11. Partial effects of predictors on literacy scores in the Estonian sample. Error bars represent the 95% confidence interval. Value “9” stands for missing or undefined responses excluding the predictor age (AGEG5LFS).

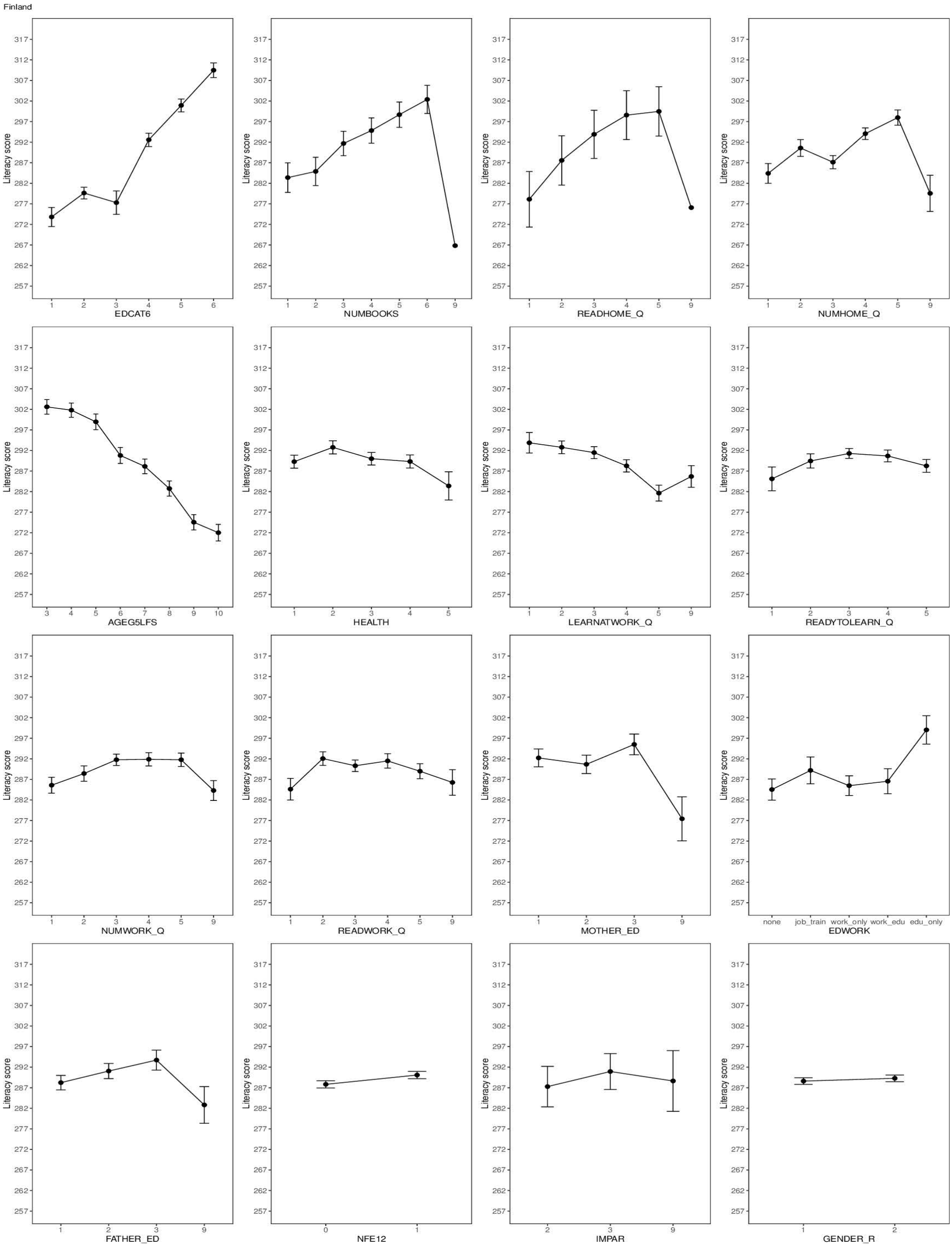

Fig 12. Partial effects of predictors on literacy scores in the Finnish sample. Error bars represent the 95% confidence interval. Value “9” stands for missing or undefined responses excluding the predictor age (AGEG5LFS). Due to increased uncertainty, some of the CIs were clipped.

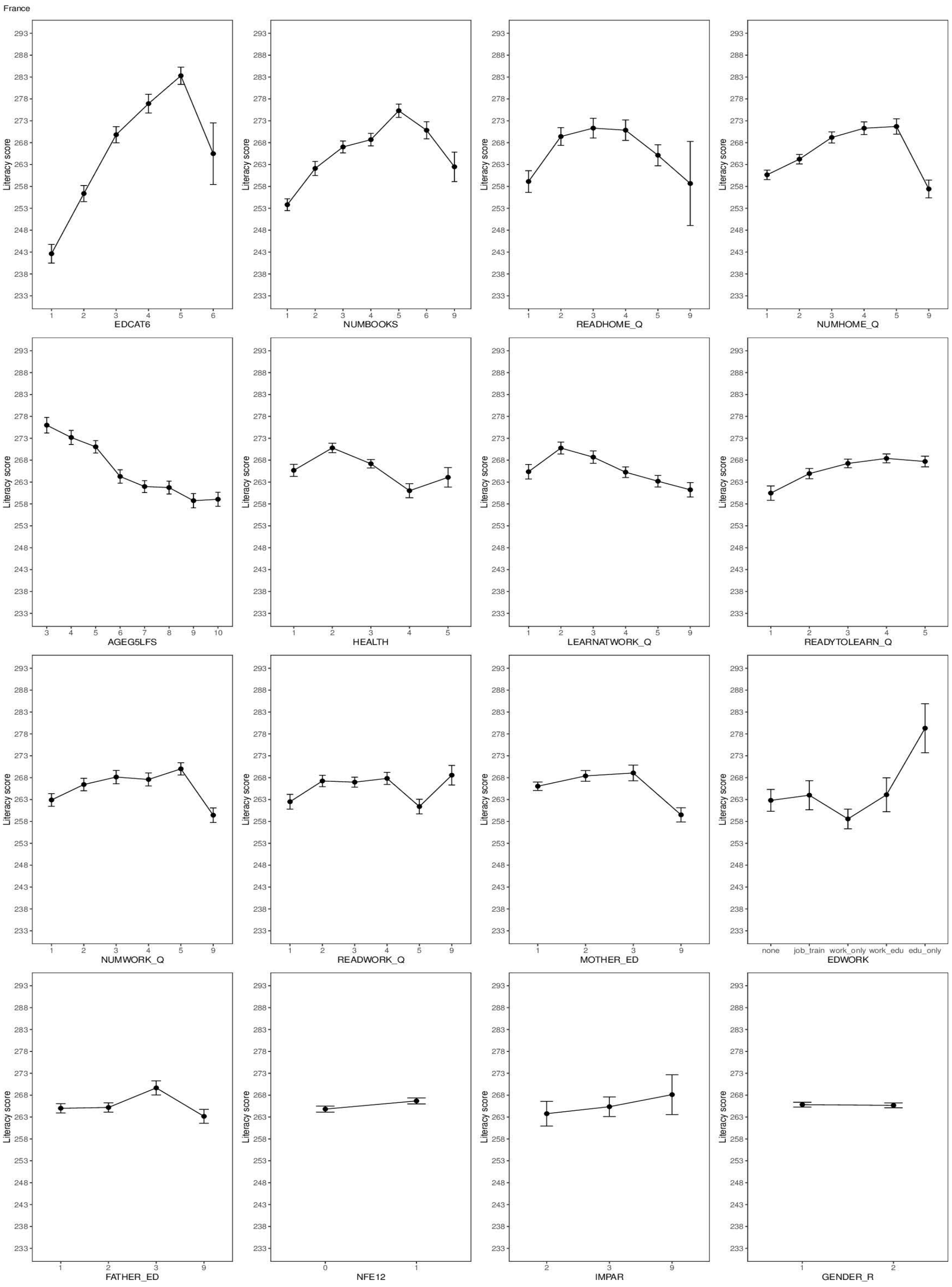

Fig 13. Partial effects of predictors on literacy scores in the French sample. Error bars represent the 95% confidence interval. Value "9" stands for missing or undefined responses excluding the predictor age (AGEG5LFS).

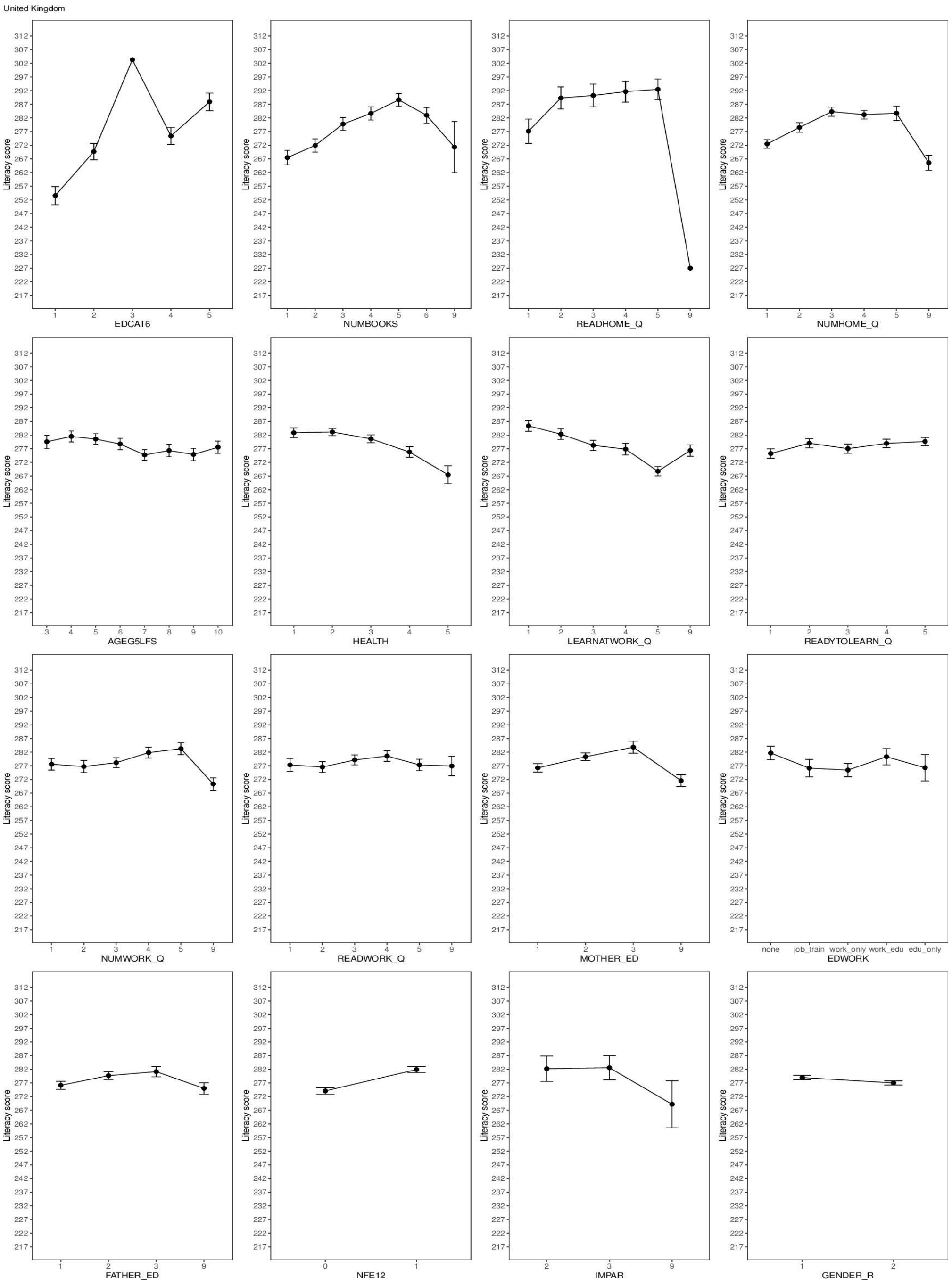

Fig 14. Partial effects of predictors on literacy scores in the British sample. Error bars represent the 95% confidence interval. Value “9” stands for missing or undefined responses excluding the predictor age (AGEG5LFS). Due to increased uncertainty, some of the CIs were clipped.

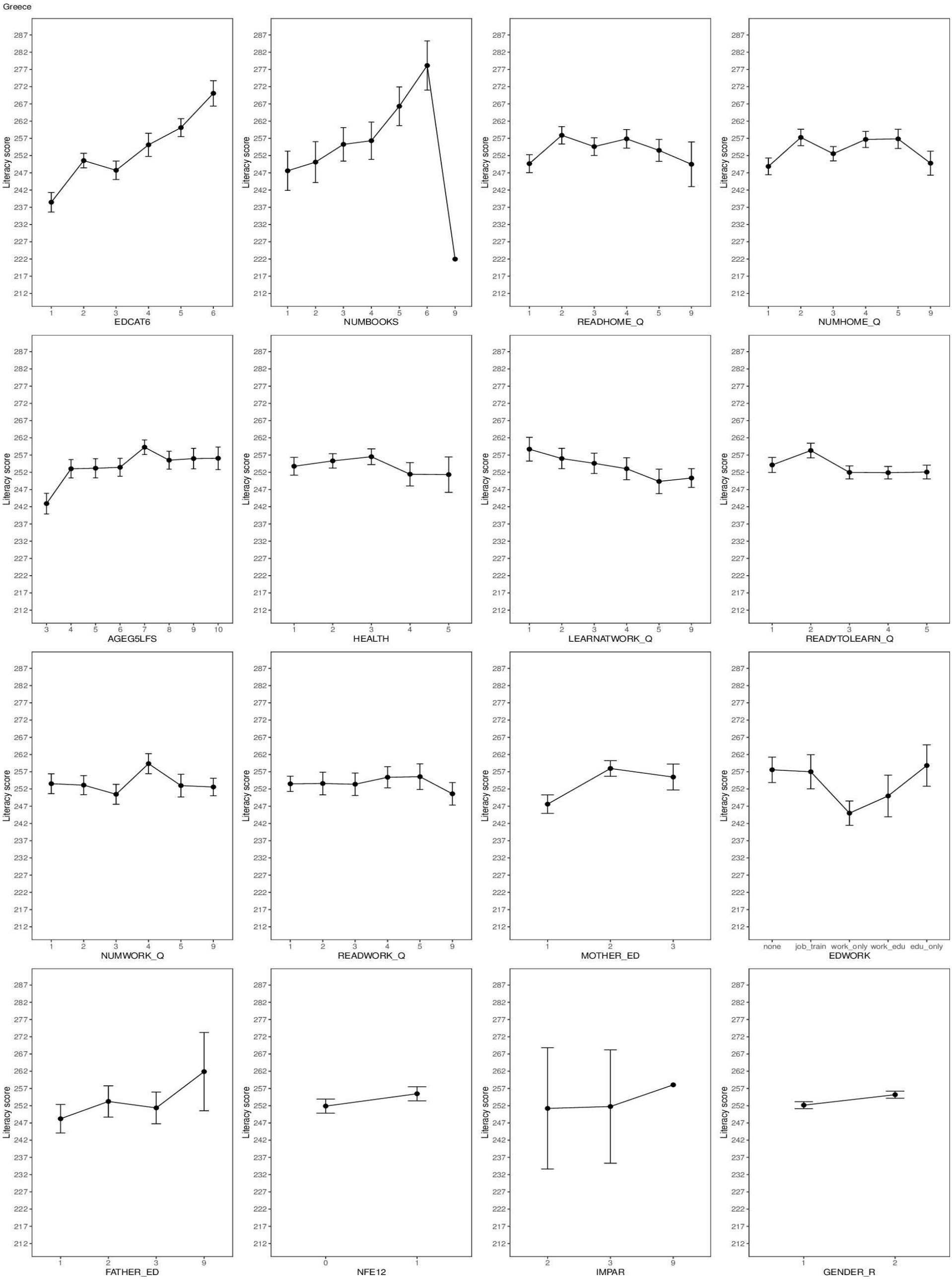

Fig 15. Partial effects of predictors on literacy scores in the Greek sample. Error bars represent the 95% confidence interval. Value "9" stands for missing or undefined responses excluding the predictor age (AGEG5LFS). Due to increased uncertainty, some of the CIs were clipped.

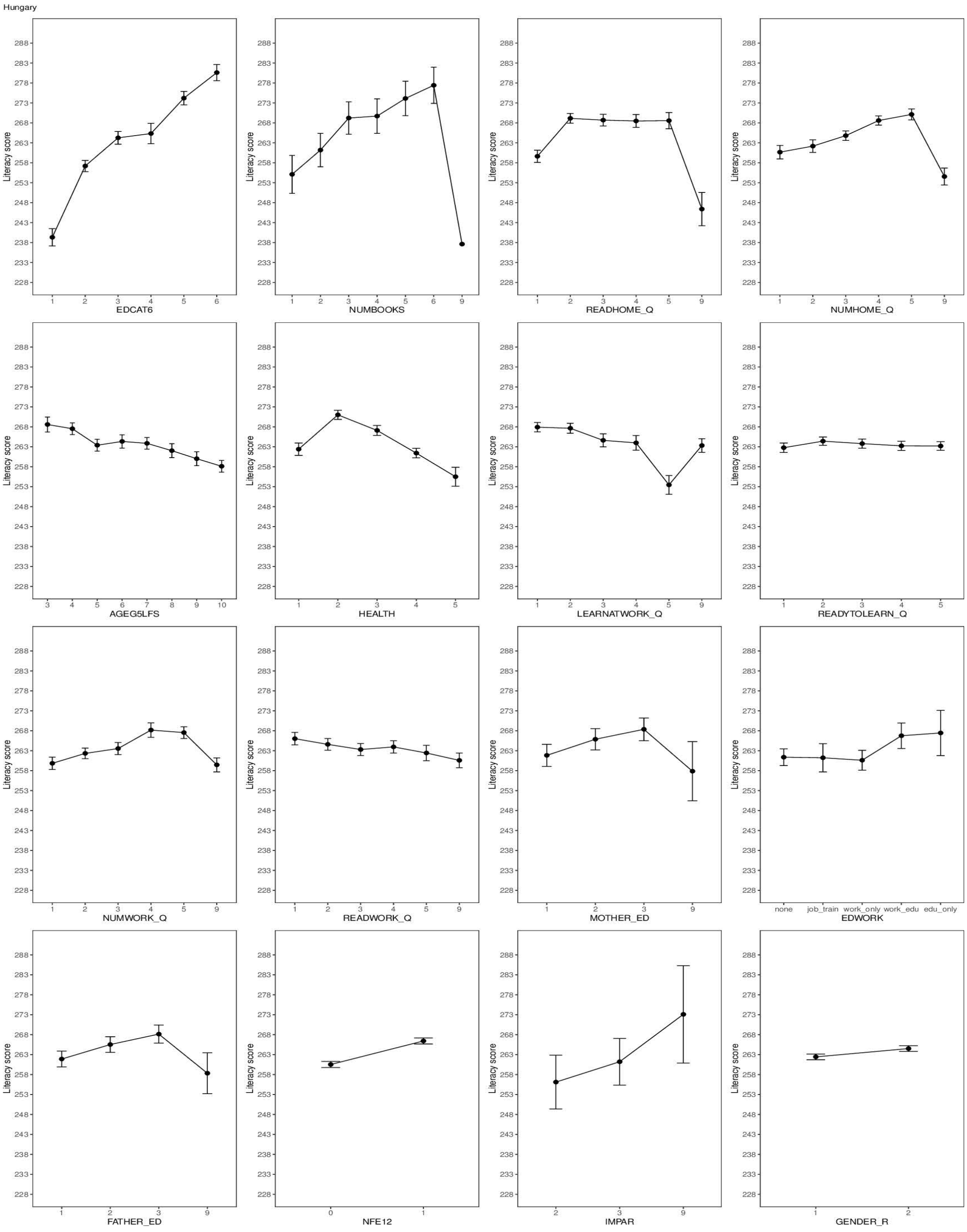

Fig 16. Partial effects of predictors on literacy scores in the Hungarian sample. Error bars represent the 95% confidence interval. Value "9" stands for missing or undefined responses excluding the predictor age (AGEG5LFS). Due to increased uncertainty, some of the CIs were clipped.

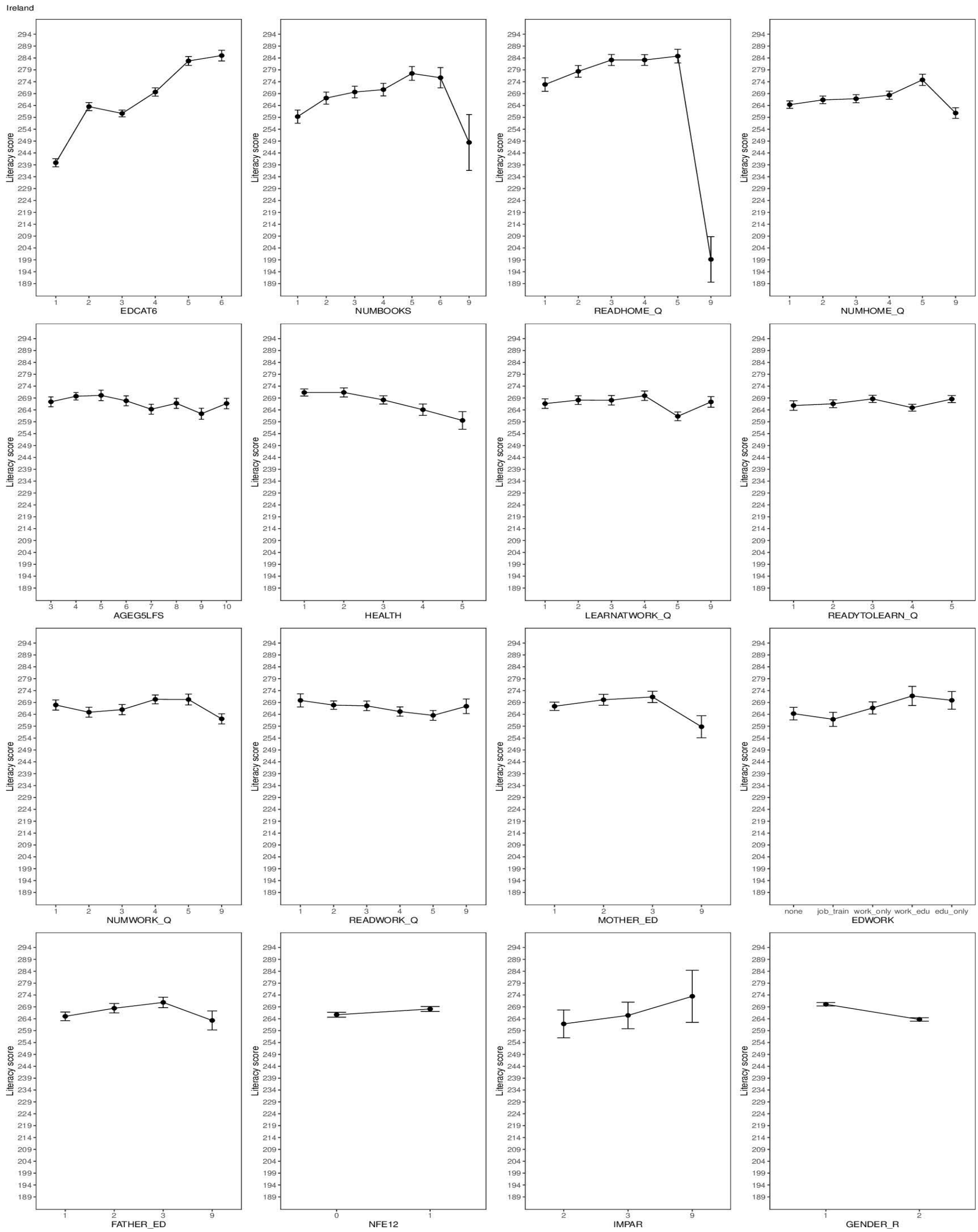

Fig 17. Partial effects of predictors on literacy scores in the Irish sample. Error bars represent the 95% confidence interval. Value "9" stands for missing or undefined responses excluding the predictor age (AGEG5LFS).

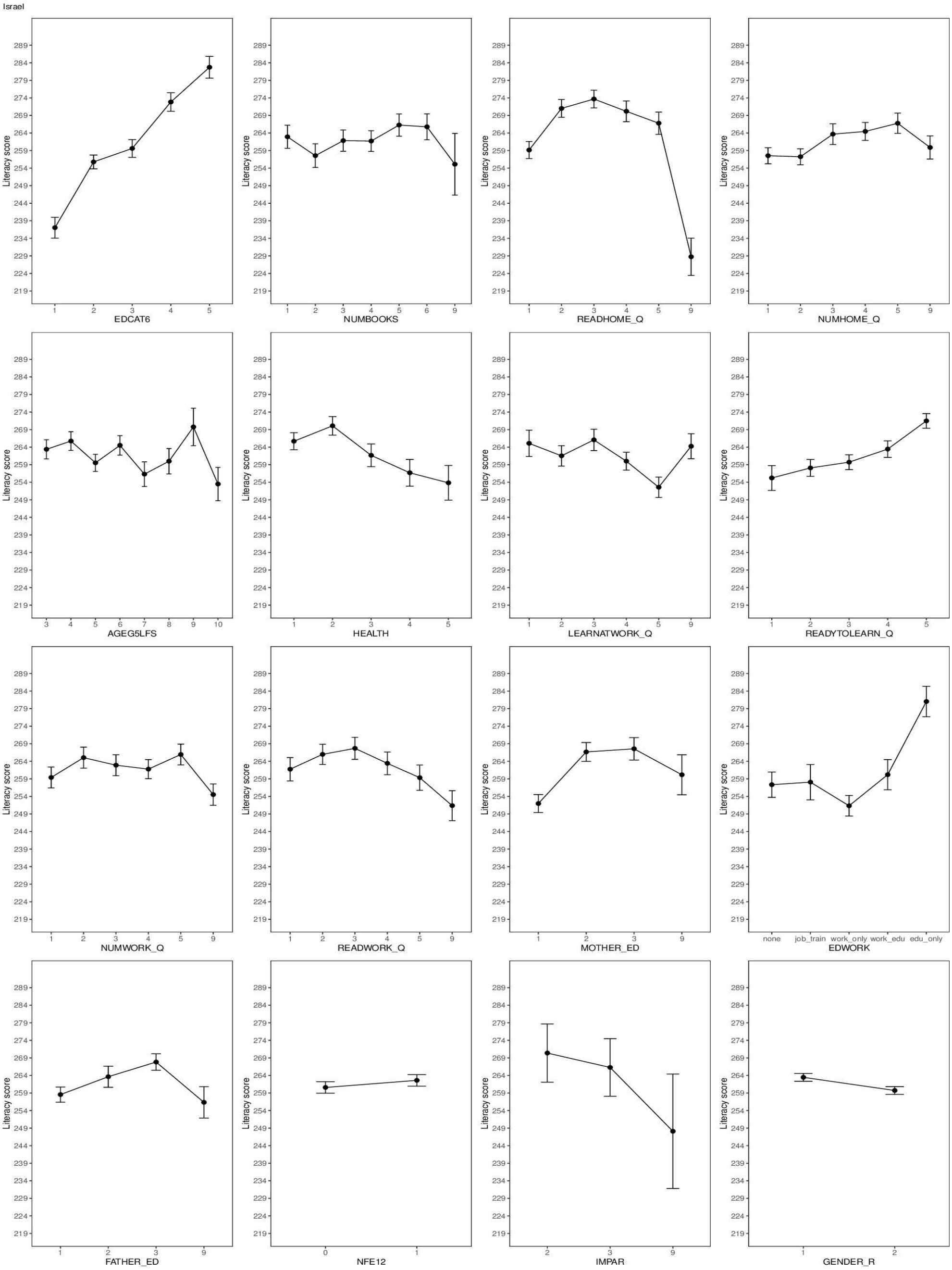

Fig 18. Partial effects of predictors on literacy scores in the Israeli sample. Error bars represent the 95% confidence interval. Value “9” stands for missing or undefined responses excluding the predictor age (AGEG5LFS).

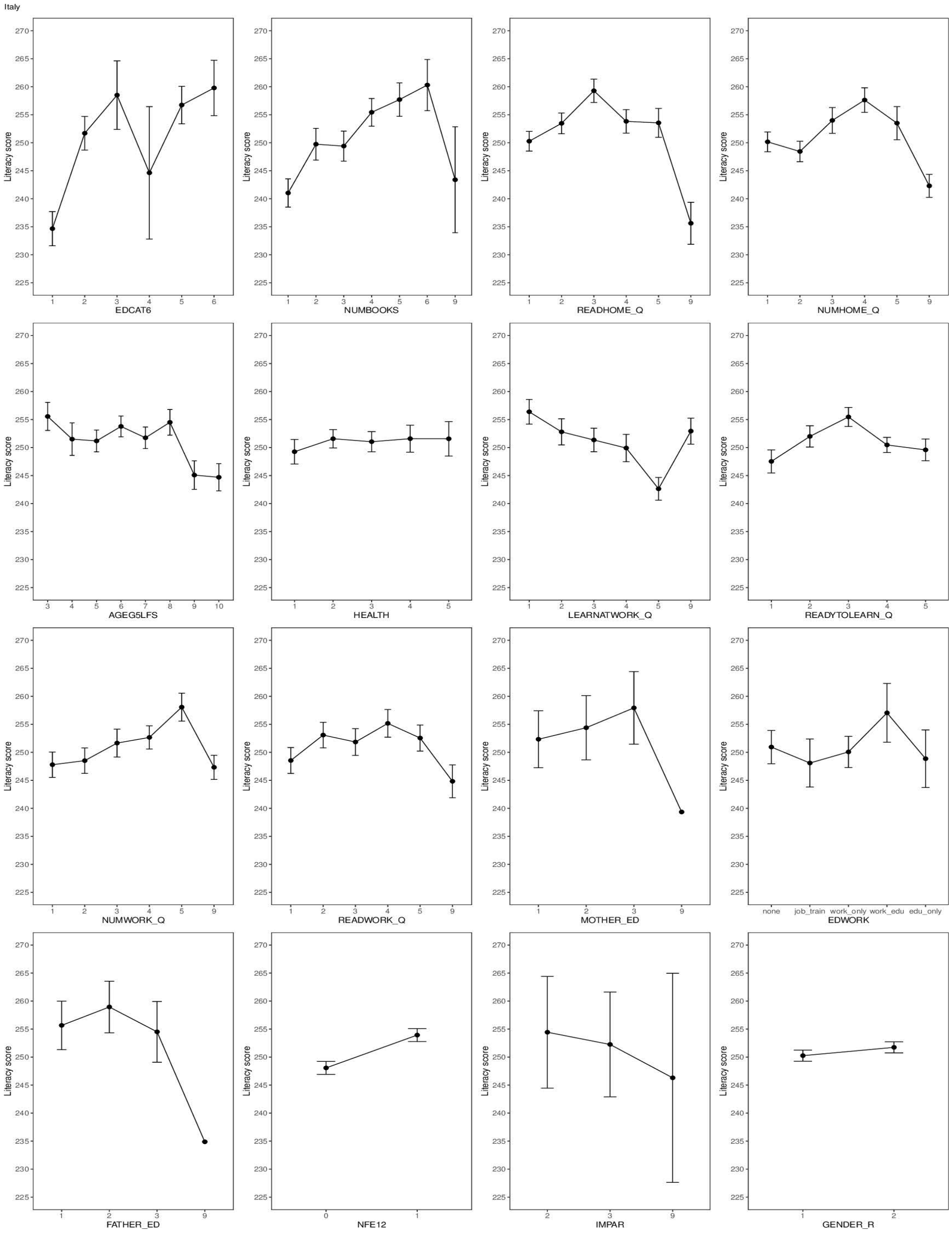

Fig 19. Partial effects of predictors on literacy scores in the Italian sample. Error bars represent the 95% confidence interval. Value "9" stands for missing or undefined responses excluding the predictor age (AGEG5LFS). Due to increased uncertainty, some of the CIs were clipped.

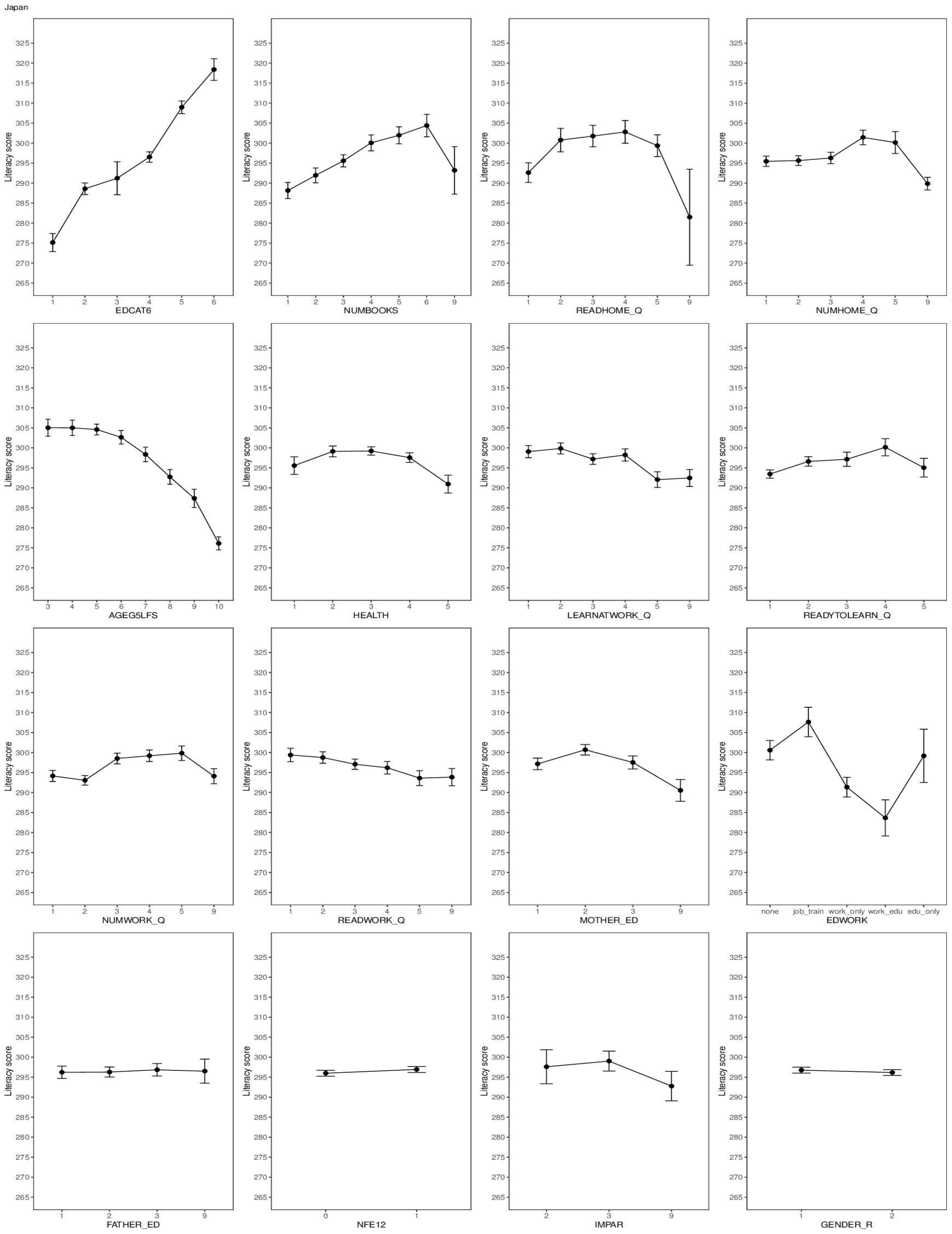

Fig 20. Partial effects of predictors on literacy scores in the Japanese sample. Error bars represent the 95% confidence interval. Value "9" stands for missing or undefined responses excluding the predictor age (AGEG5LFS).

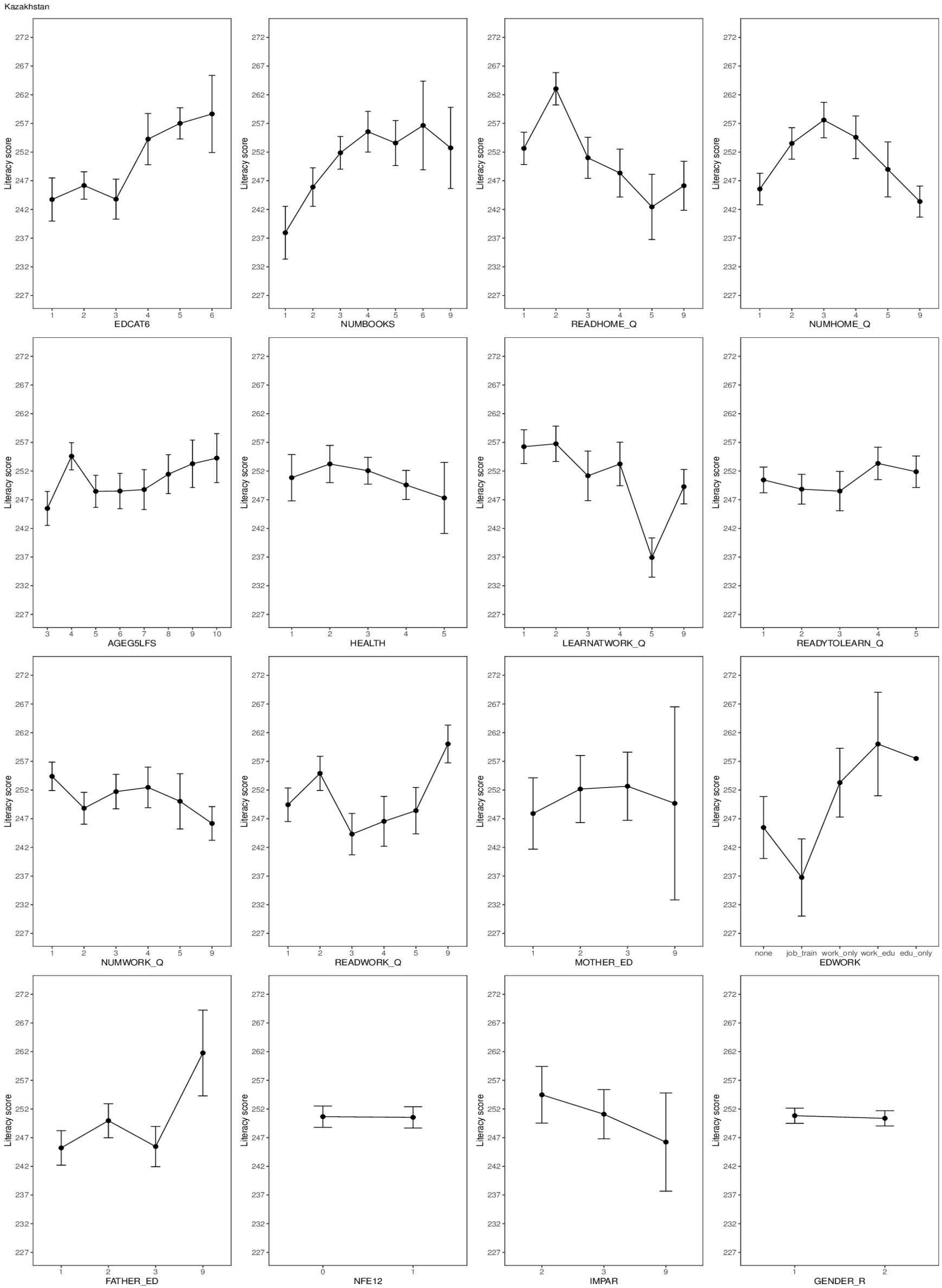

Fig 21. Partial effects of predictors on literacy scores in the Kazakhstani sample. Error bars represent the 95% confidence interval. Value "9" stands for missing or undefined responses excluding the predictor age (AGEG5LFS).

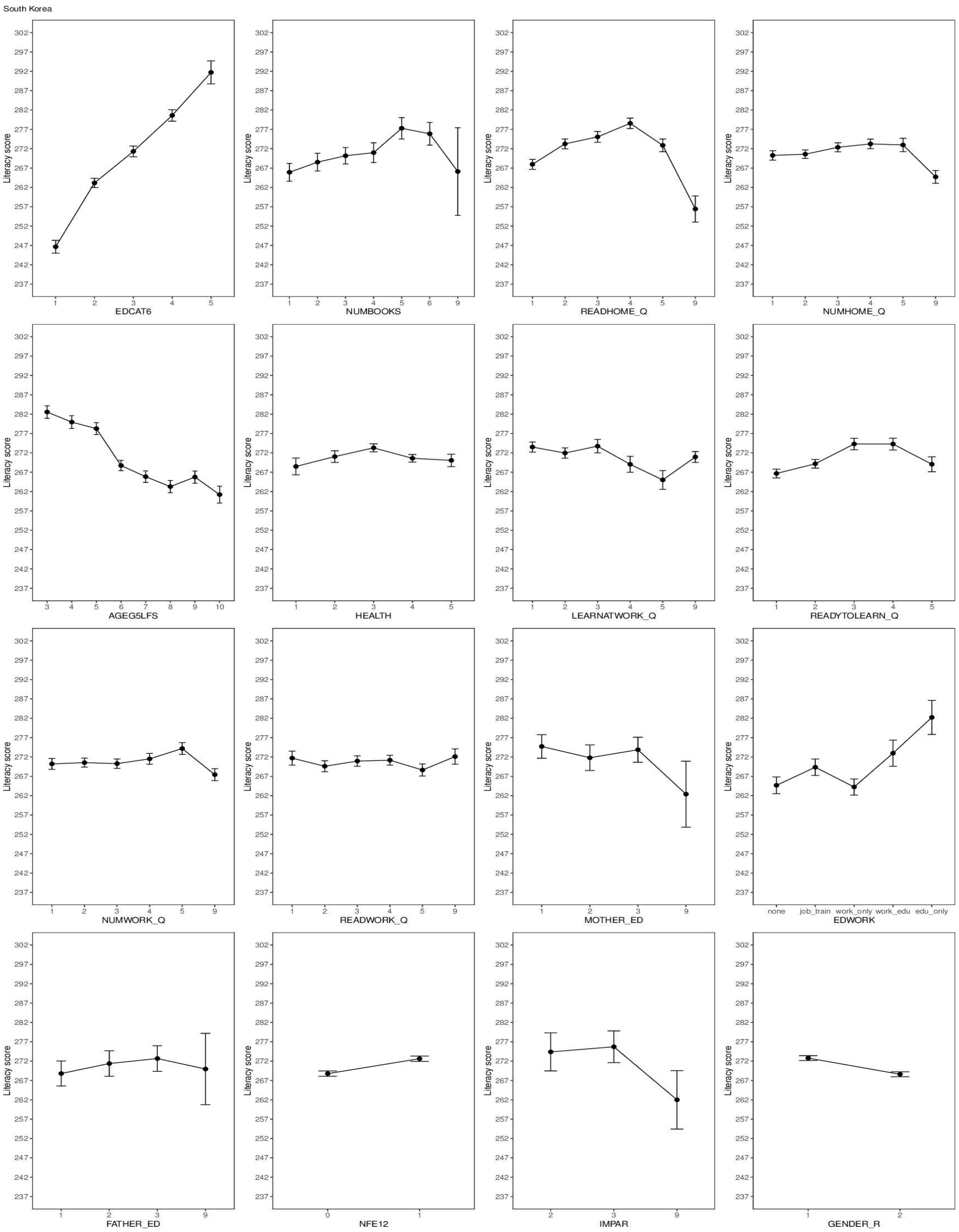

Fig 22. Partial effects of predictors on literacy scores in the South Korean sample. Error bars represent the 95% confidence interval. Value "9" stands for missing or undefined responses excluding the predictor age (AGEG5LFS).

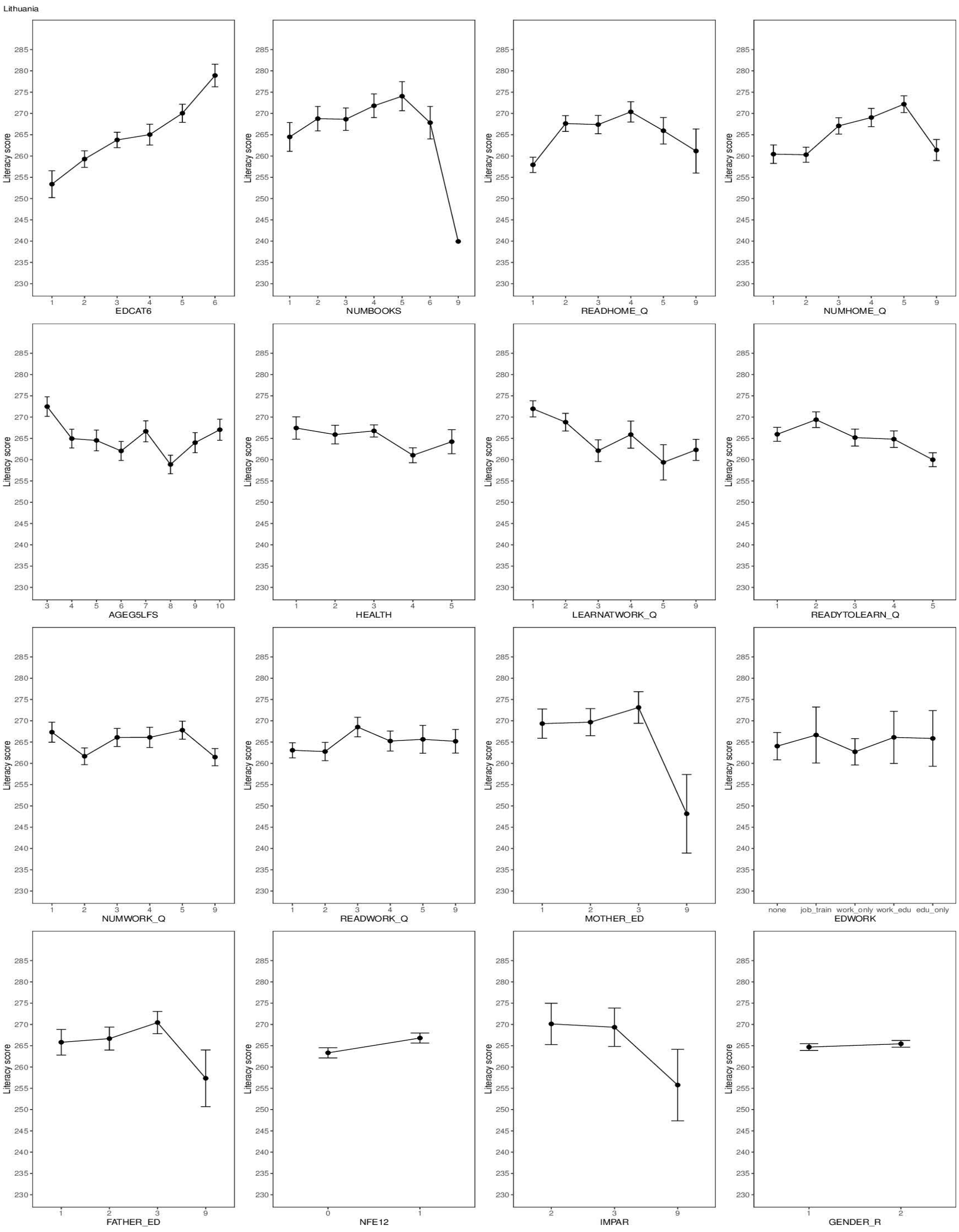

Fig 23. Partial effects of predictors on literacy scores in the Lithuanian sample. Error bars represent the 95% confidence interval. Value "9" stands for missing or undefined responses excluding the predictor age (AGEG5LFS). Due to increased uncertainty, some of the CIs were clipped.

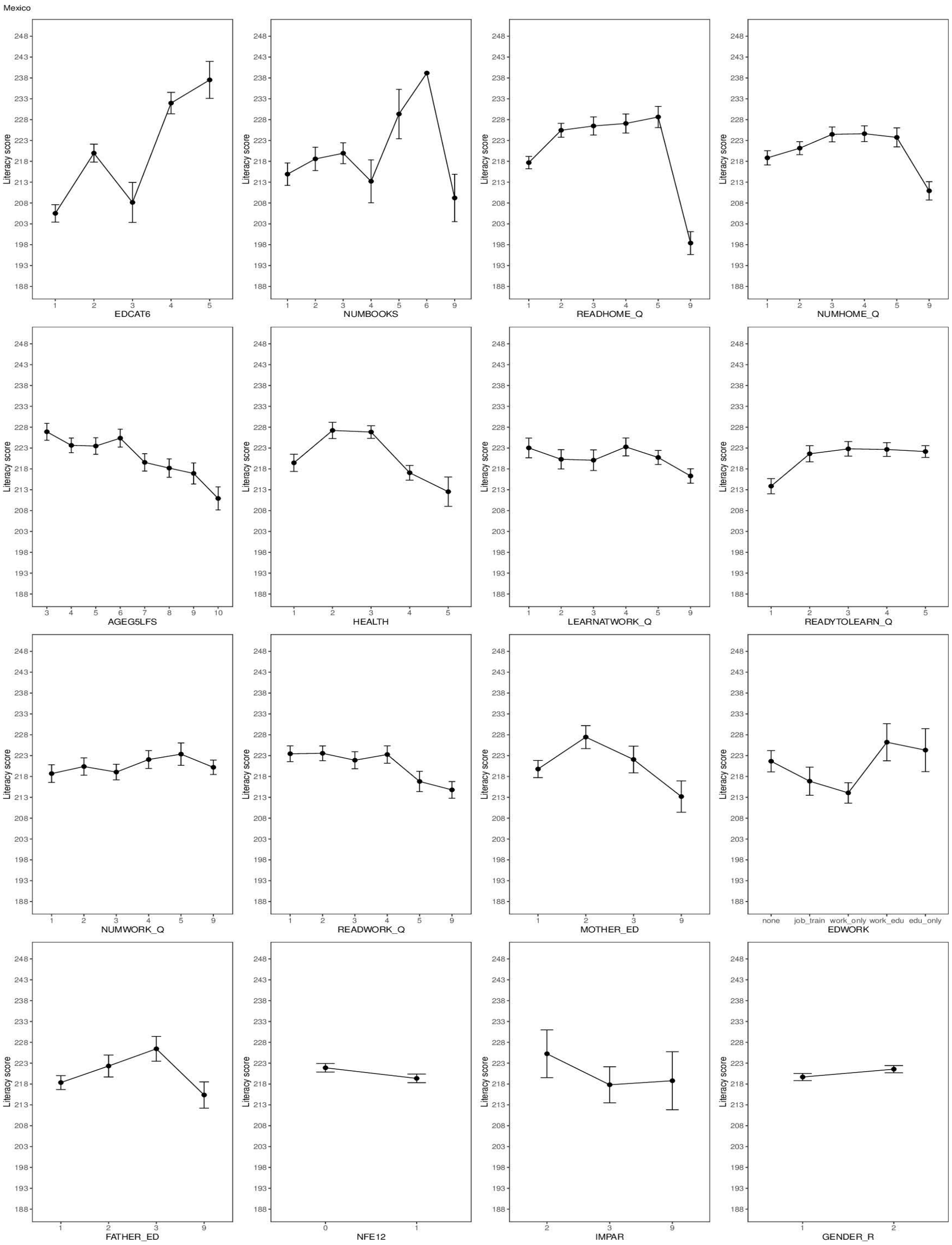

Fig 24. Partial effects of predictors on literacy scores in the Mexican sample. Error bars represent the 95% confidence interval. Value "9" stands for missing or undefined responses excluding the predictor age (AGEG5LFS).

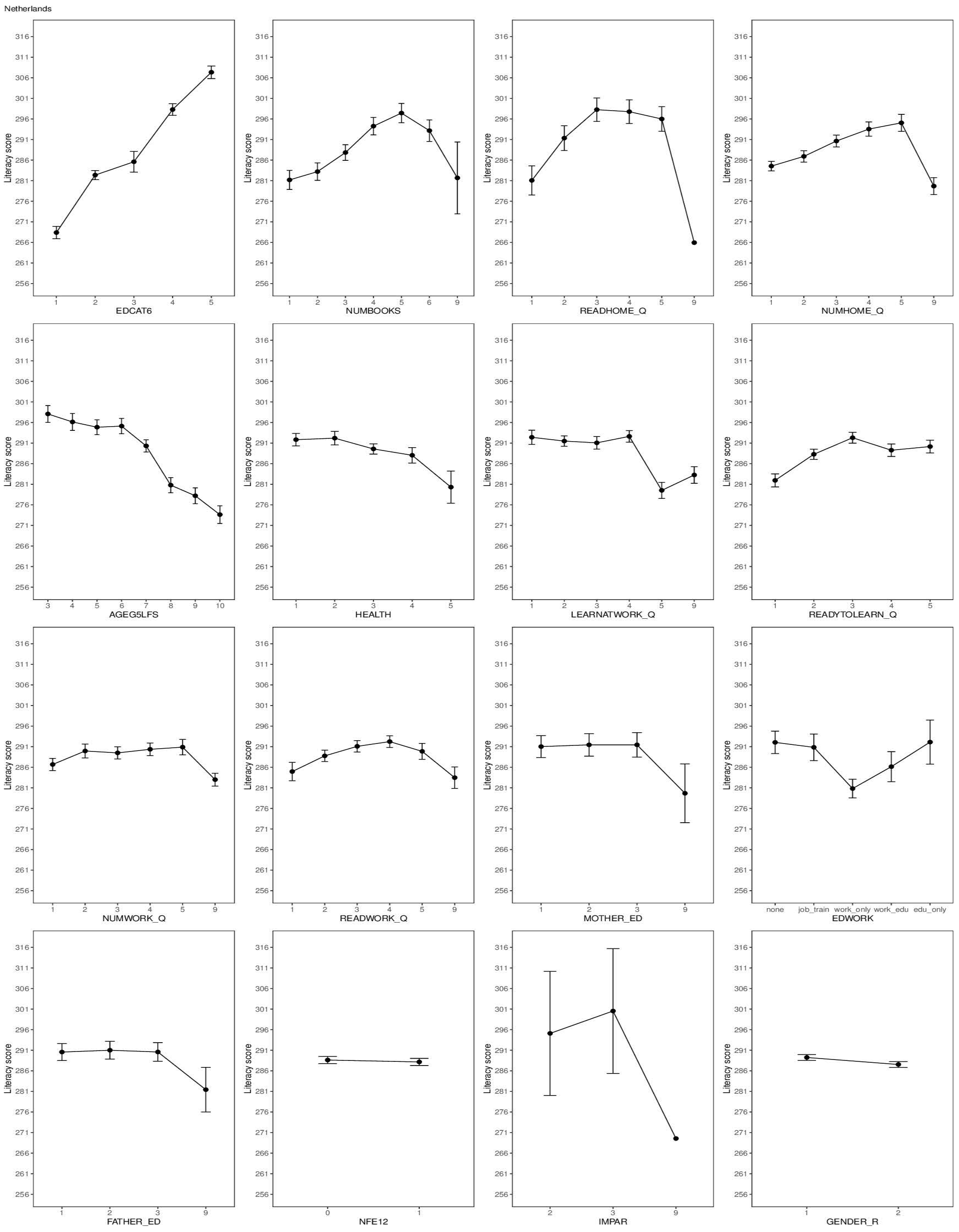

Fig 25. Partial effects of predictors on literacy scores in the Dutch sample. Error bars represent the 95% confidence interval. Value "9" stands for missing or undefined responses excluding the predictor age (AGEG5LFS). Due to increased uncertainty, some of the CIs were clipped.

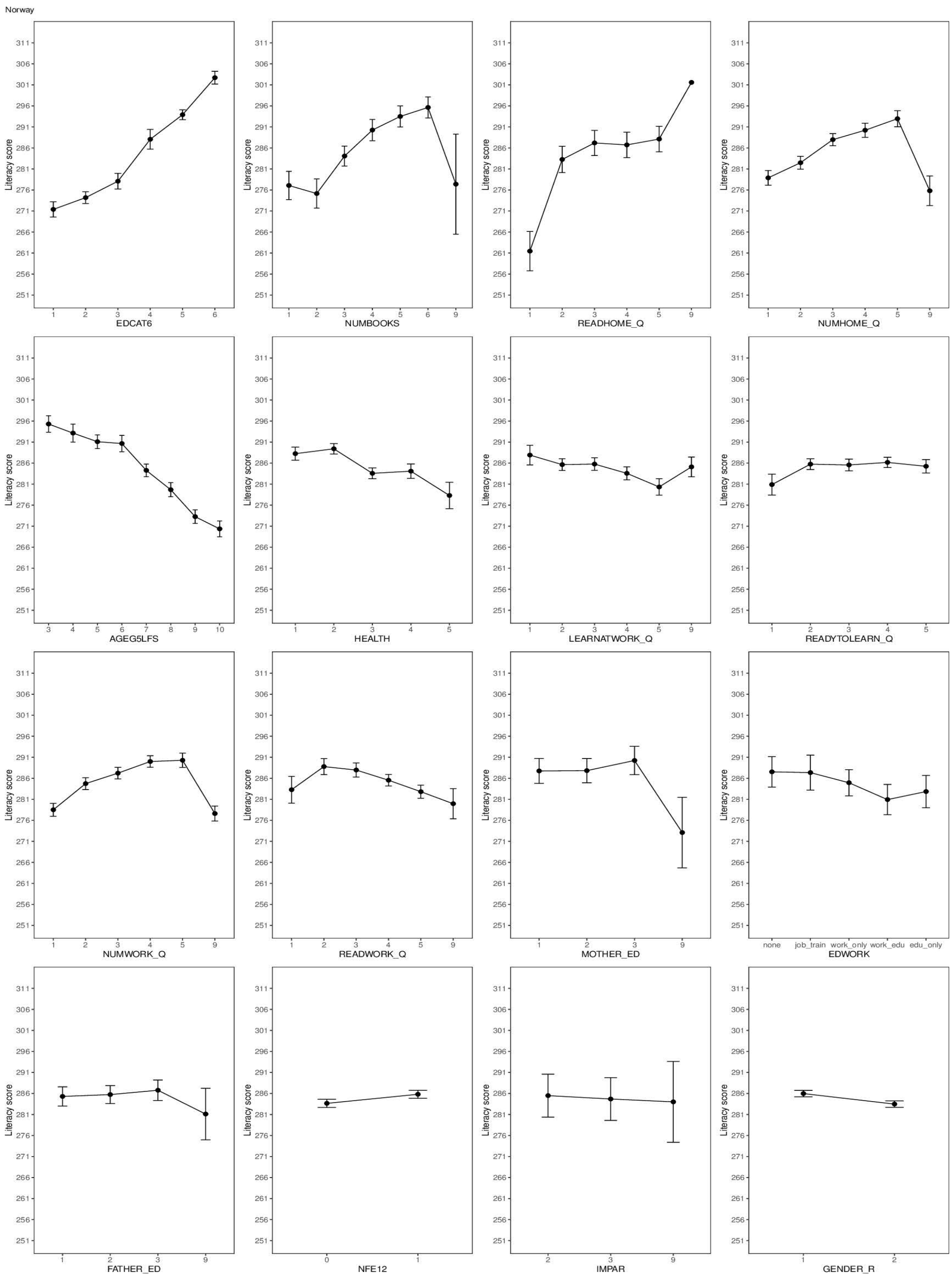

Fig 26. Partial effects of predictors on literacy scores in the Norwegian sample. Error bars represent the 95% confidence interval. Value “9” stands for missing or undefined responses excluding the predictor age (AGEG5LFS).

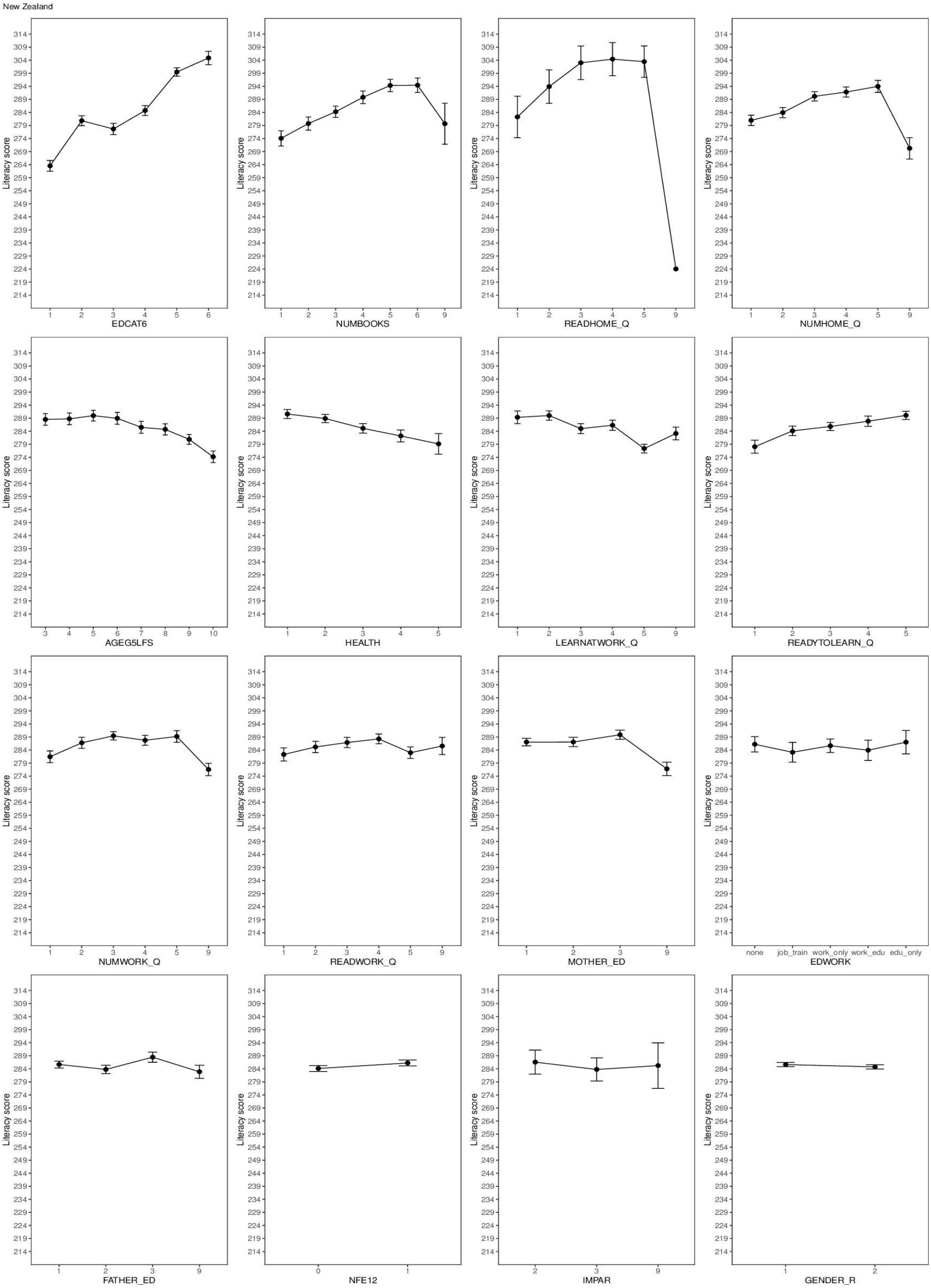

Fig 27. Partial effects of predictors on literacy scores in the New Zealand sample. Error bars represent the 95% confidence interval. Value "9" stands for missing or undefined responses excluding the predictor age (AGEG5LFS). Due to increased uncertainty, some of the CIs were clipped.

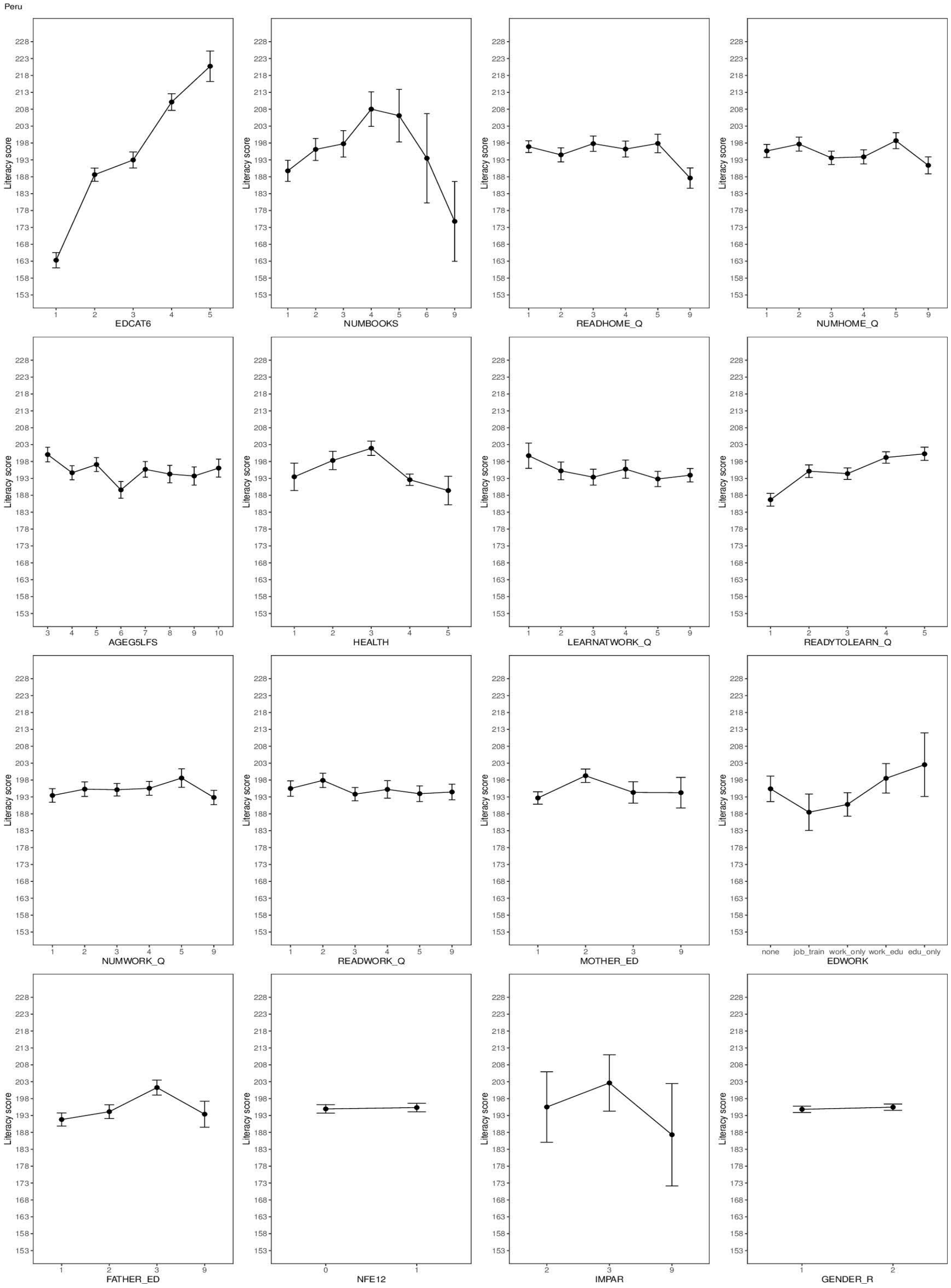

Fig 28. Partial effects of predictors on literacy scores in the Peruvian sample. Error bars represent the 95% confidence interval. Value "9" stands for missing or undefined responses excluding the predictor age (AGEG5LFS).

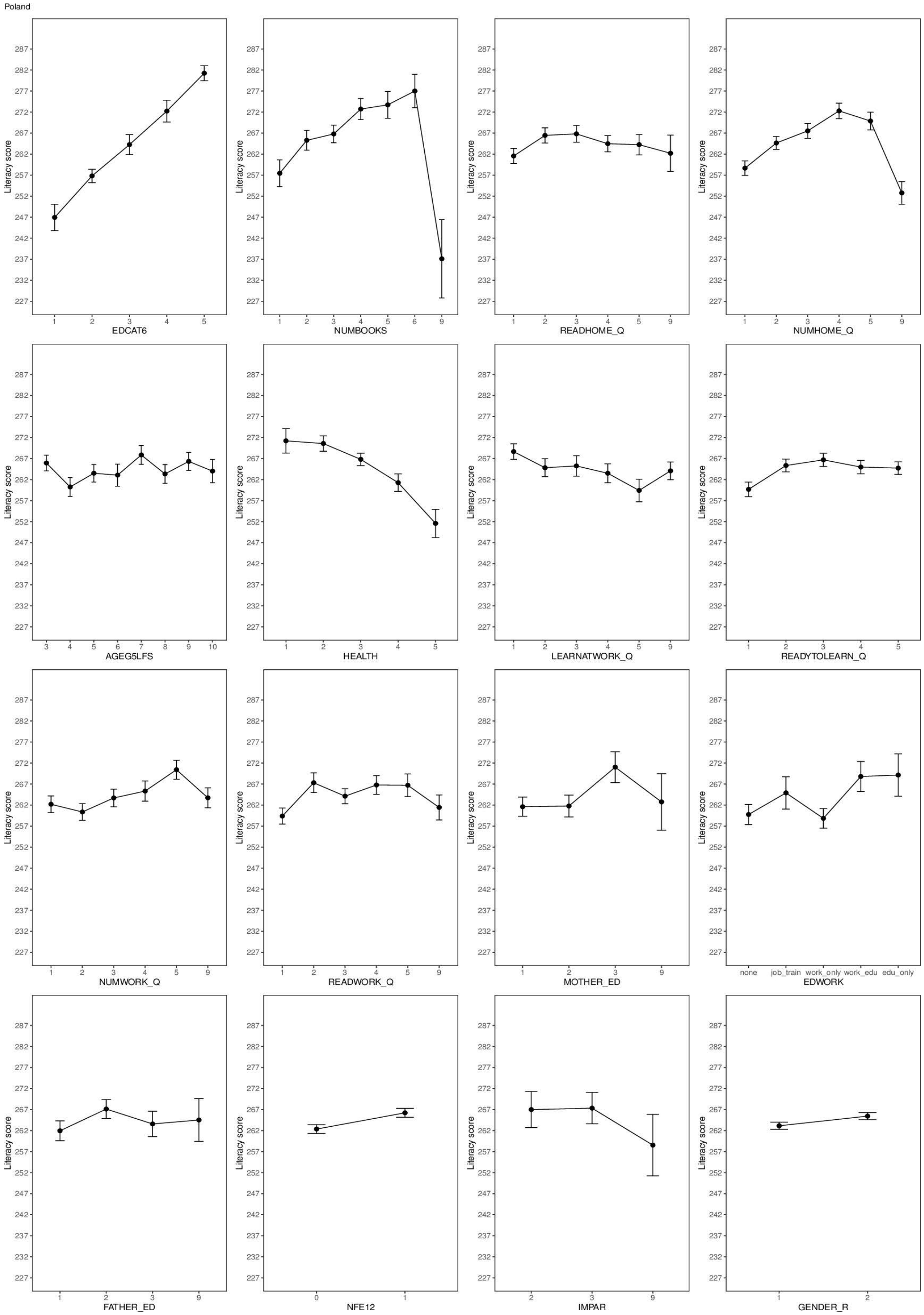

Fig 29. Partial effects of predictors on literacy scores in the Polish sample. Error bars represent the 95% confidence interval. Value "9" stands for missing or undefined responses excluding the predictor age (AGEG5LFS).

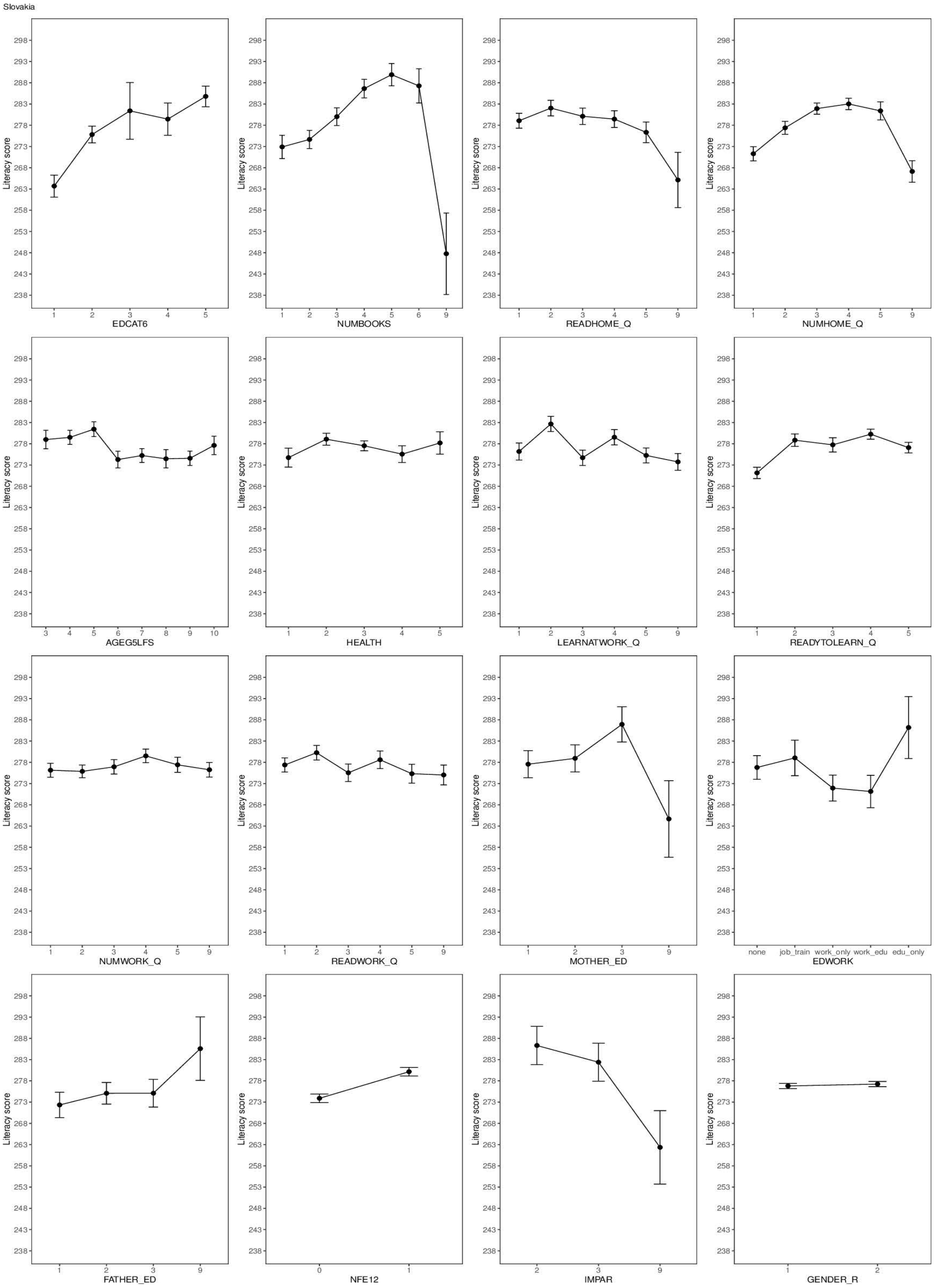

Fig 30. Partial effects of predictors on literacy scores in the Slovak sample. Error bars represent the 95% confidence interval. Value “9” stands for missing or undefined responses excluding the predictor age (AGEG5LFS).

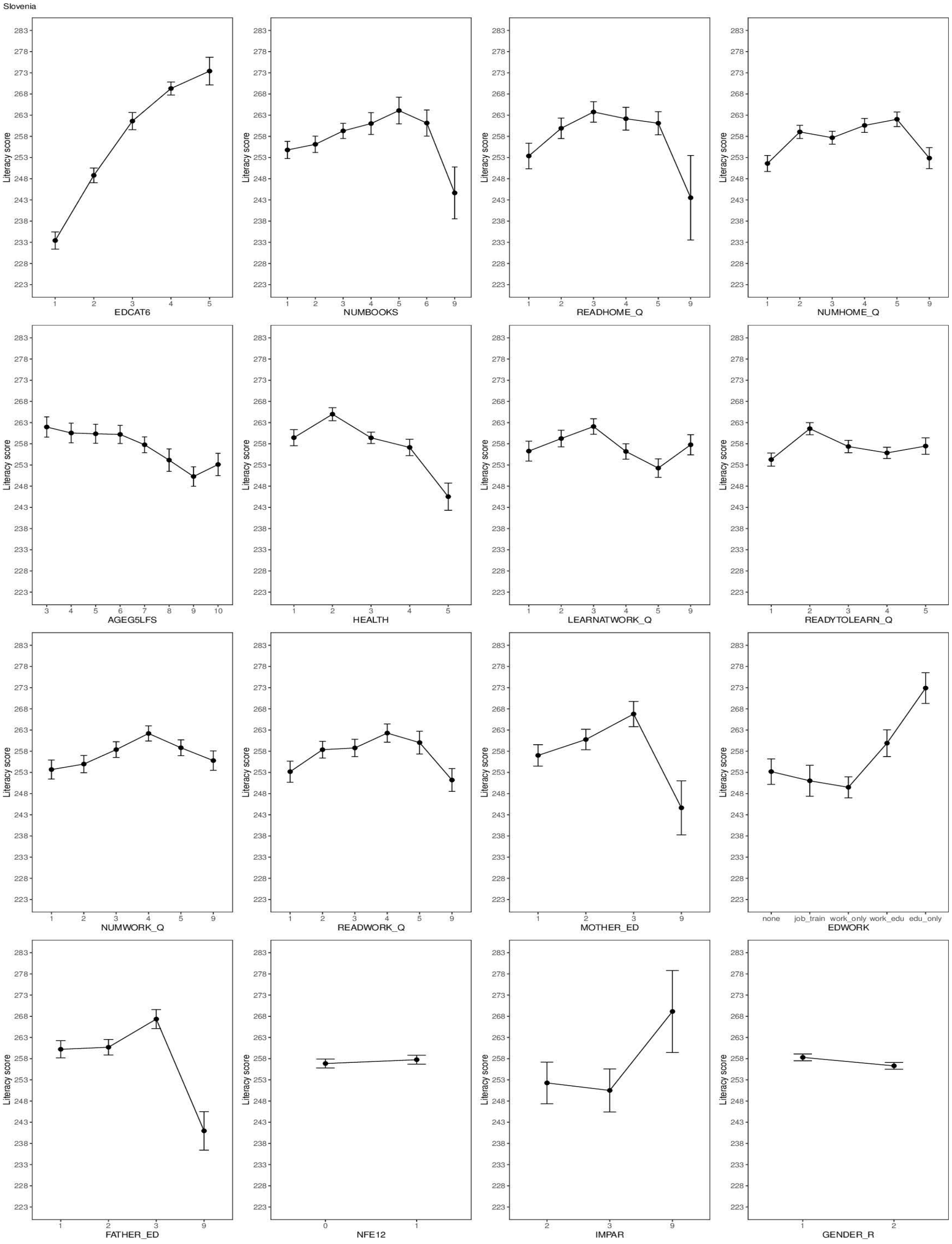

Fig 31. Partial effects of predictors on literacy scores in the Slovenian sample. Error bars represent the 95% confidence interval. Value “9” stands for missing or undefined responses excluding the predictor age (AGEG5LFS).

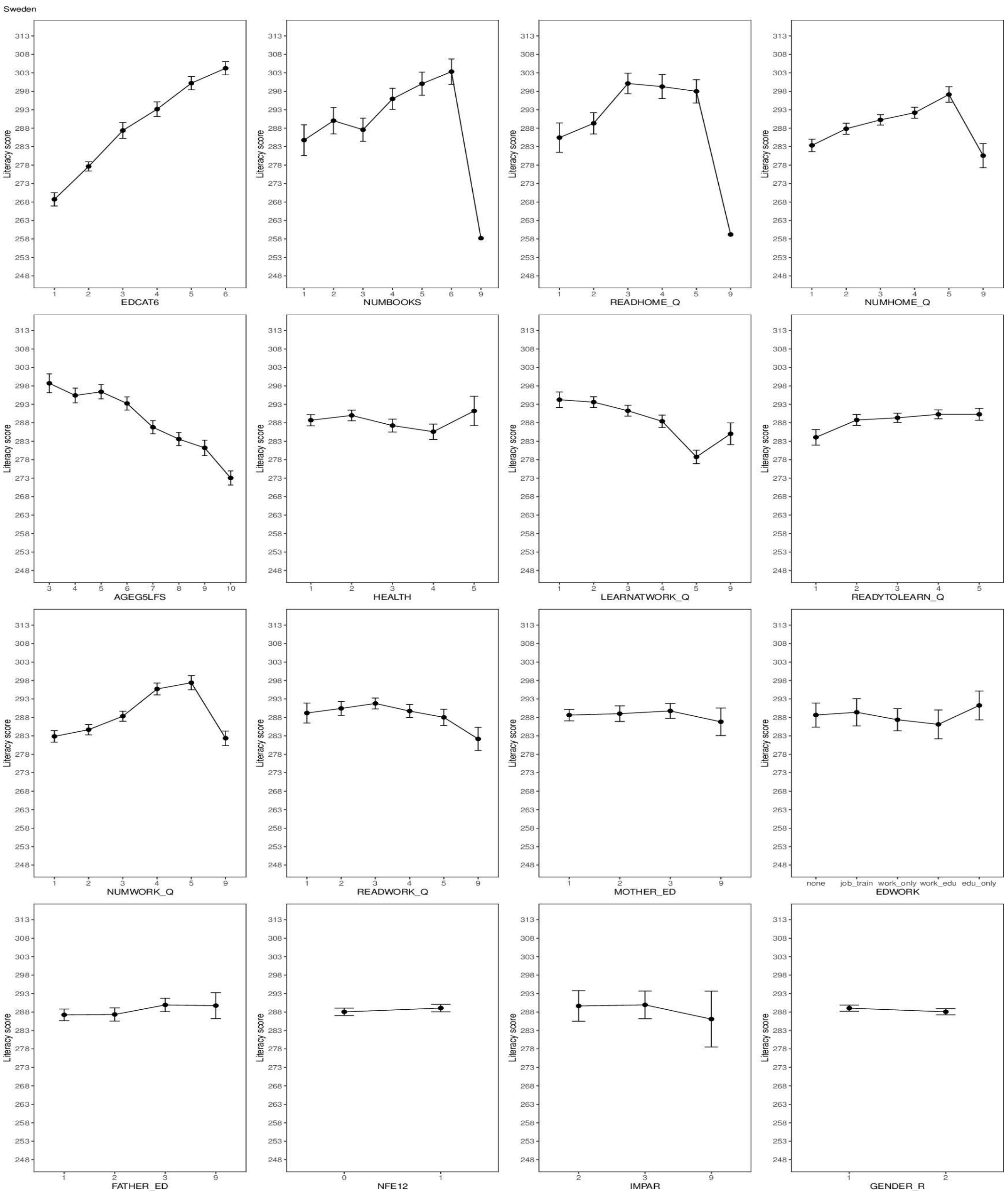

Fig 32. Partial effects of predictors on literacy scores in the Swedish sample. Error bars represent the 95% confidence interval. Value "9" stands for missing or undefined responses excluding the predictor age (AGEG5LFS). Due to increased uncertainty, some of the CIs were clipped.

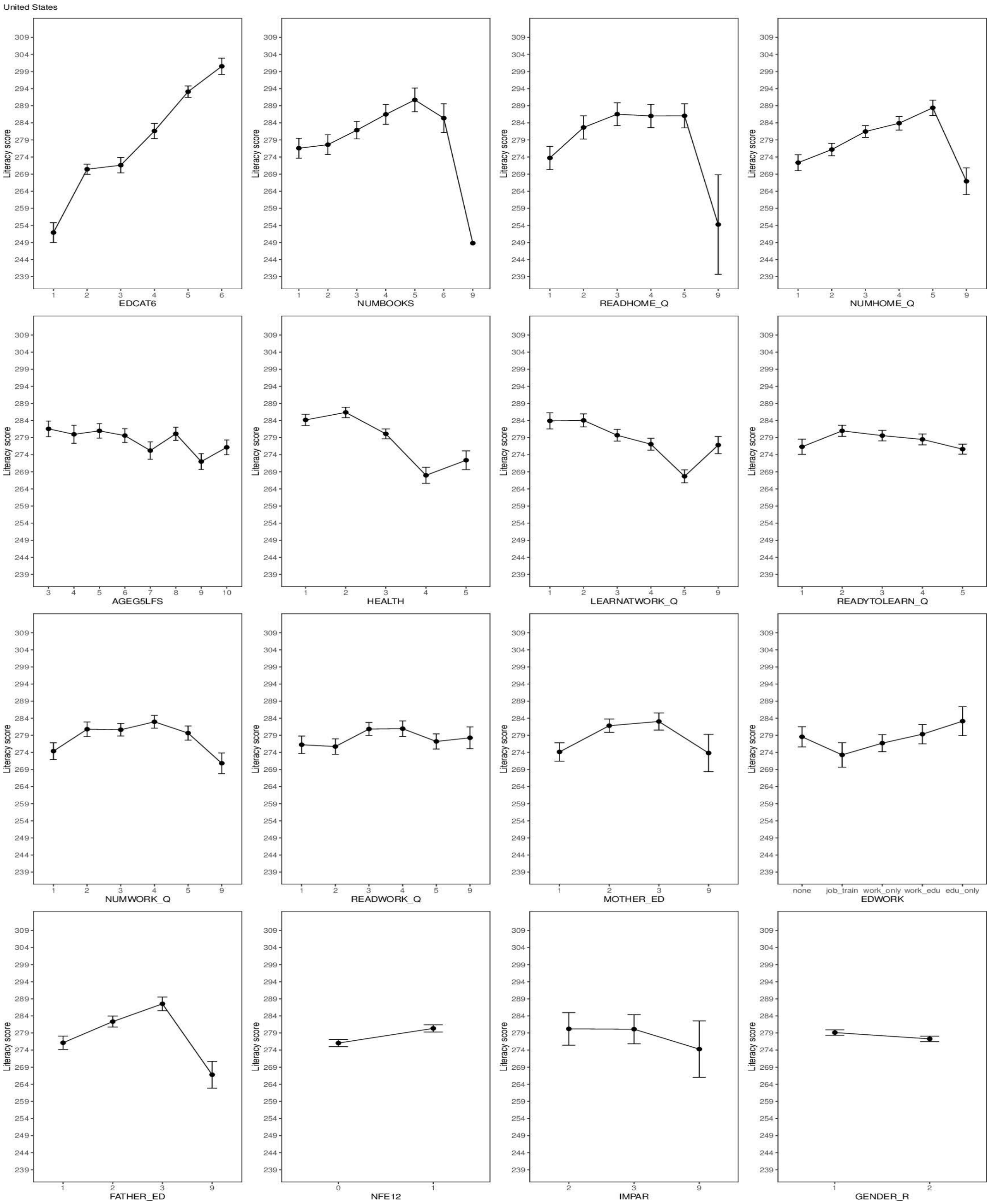

Fig 33. Partial effects of predictors on literacy scores in the American sample. Error bars represent the 95% confidence interval. Value "9" stands for missing or undefined responses excluding the predictor age (AGEG5LFS). Due to increased uncertainty, some of the CIs were clipped.

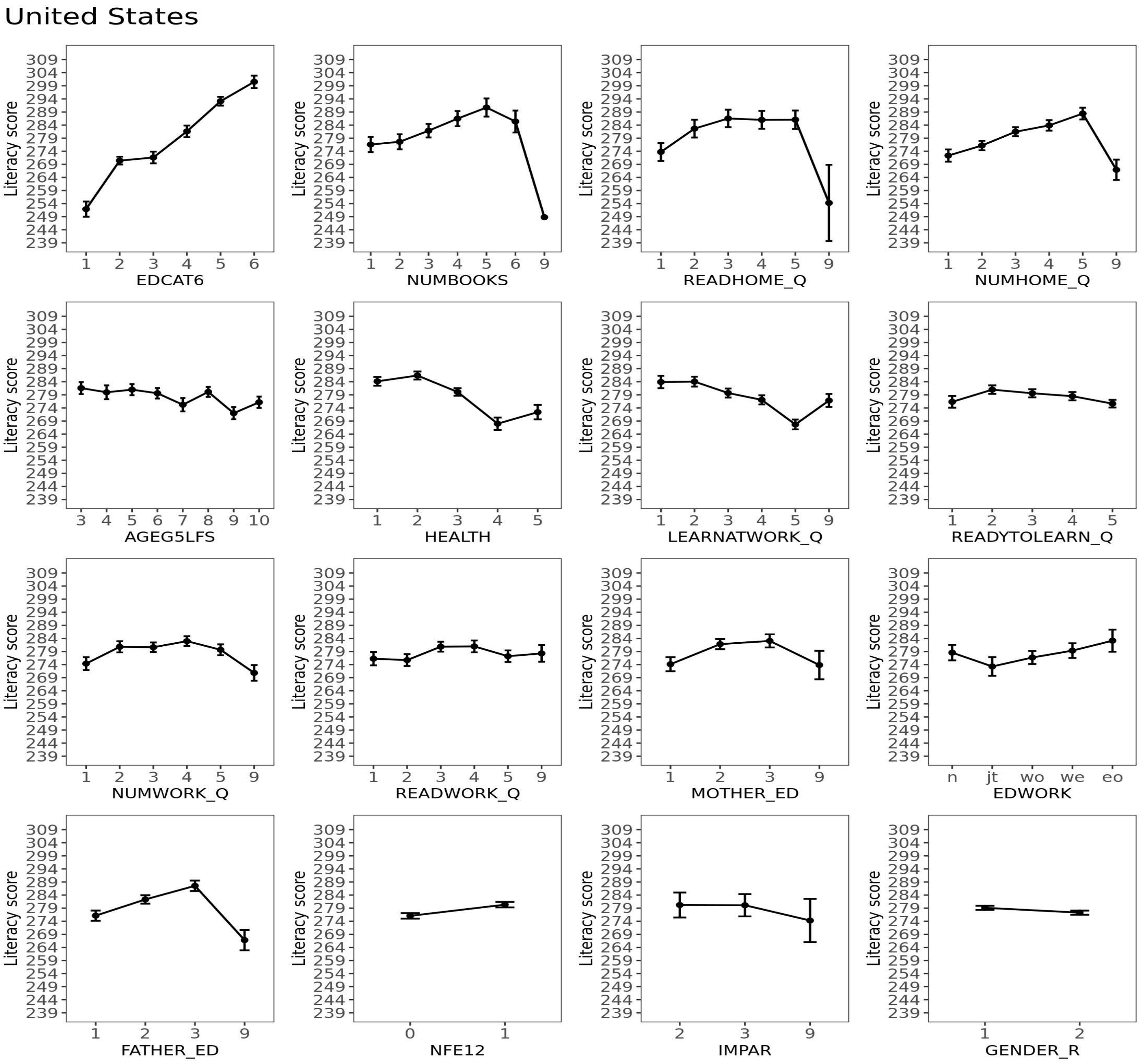

3 S3: Description of the derived variables

We provide for each of the derived variables that were used in this study their corresponding reference variables along with the exact question used in the survey. This information is presented separately for each of the derived variables in the tables below.

Table 4. The reference variables for the derived variable NUMHOM

Reference variable	Question
H_Q03b	In everyday life, how often do you usually... calculate prices, costs or budgets?
H_Q03c	use or calculate fractions, decimals or percentages?
H_Q03d	use a calculator - either hand-held or computer based?
H_Q03f	In everyday life, how often do you usually... prepare charts, graphs or tables
H_Q03g	use simple algebra or formulas?
H_Q03h	use more advanced math or statistics such as calculus, complex algebra, trigonometry or use of regression techniques?

Table 5. The reference variables for the derived variable READHOME

Reference variable	Question
H_Q01a	In everyday life, how often do you usually...read directions or instructions?
H_Q01b	read letters, memos or e-mails?
H_Q01c	read articles in newspapers, magazines or newsletters?
H_Q01d	read articles in professional journals or scholarly publications?
H_Q01e	In everyday life, how often do you usually... read books, fiction or non-fiction?
H_Q01f	read manuals or reference materials?
H_Q01g	read bills, invoices, bank statements or other financial
H_Q01h	read diagrams, maps, or schematics?

Table 6. The reference variables for the derived variable LEARNATWORK

Reference variable	Question
D_Q13a	In your own job, how often do you learn new work-related things from co-workers or supervisors?
D_Q13b	How often does your job involve learning-by-doing from the tasks you perform?
D_Q13c	How often does your job involve keeping up to date with new products or services?
H_Q03g	use simple algebra or formulas?
H_Q03h	use more advanced math or statistics such as calculus, complex algebra, trigonometry or use of regression techniques?

Table 7. The reference variables for the derived variable NUMWORK

Reference variable	Question
G_Q03b	In your Job/Lastjob, how often Do/Did you usually... calculate prices, costs or budgets?
G_Q03c	use or calculate fractions, decimals or percentages?
G_Q03d	use a calculator - either hand-held or computer based?
G_Q03f	In your Job/Lastjob, how often Do/Did you usually... prepare charts, graphs or tables?
G_Q03g	use simple algebra or formulas?
G_Q03h	use more advanced math or statistics such as calculus, complex algebra, trigonometry or use of regression techniques?

Table 8. The reference variables for the derived variable READWORK

Reference variable	Question
G_Q01a	In your Job/Lastjob, how often Do/Did you usually... read directions or instructions?
G_Q01b	read letters, memos or e-mails?
G_Q01c	read articles in newspapers, magazines or newsletters?
G_Q01d	read articles in professional journals or scholarly publications?
G_Q01e	In your Job/Lastjob, how often Do/Did you usually... read books?
G_Q01f	read manuals or reference materials?
G_Q01g	read bills, invoices, bank statements or other financial statements?
G_Q01h	read diagrams, maps or schematics?
